# Osteoimmune senescence in aging-related bone diseases

**DOI:** 10.3389/fimmu.2026.1892334

**Published:** 2026-07-15

**Authors:** Huali Li, Yingli Yang, Huanle Zhu

**Affiliations:** 1 Department of Radiology, Sir Run Run Shaw Hospital, Zhejiang University School of Medicine, Hangzhou, China; 2 Department of Pharmacy, Women’s Hospital, School of Medicine Zhejiang University, Hangzhou, China

**Keywords:** aging-related bone diseases, cellular senescence, inflammaging, osteoimmune senescence, senotherapeutics

## Abstract

Aging-related bone diseases are increasingly recognized as disorders in which uncoupled bone remodeling remains central but is substantially modified by immune-skeletal interactions. This review consolidates current evidence for osteoimmune senescence, a framework that links senescent skeletal cells, aging immune compartments, chronic SASP signaling, impaired immune clearance, and marrow-niche deterioration to reduced bone strength and repair. Osteoporosis, rheumatoid arthritis-associated erosion, osteoarthritis, periodontal bone loss, and delayed fracture healing are clinically distinct diseases, but they may share overlapping senescence-associated osteoimmune mechanisms while retaining disease-specific endotypes. Senescent osteocytes, osteoblast-lineage cells, and bone marrow stromal cells can increase RANKL/OPG imbalance, suppress osteogenesis, and reshape immune recruitment, whereas remodeled T cells, macrophages, neutrophils, and NK-cell surveillance pathways modify osteoclastogenesis and repair resolution. We also discuss gut-derived metabolites and imaging phenotypes as clinically important but non-specific readouts of this biology. DXA, HR-pQCT, MRI, CBCT, and molecular imaging cannot identify senescent cells directly, but they can anchor molecular hypotheses to tissue-level deterioration. Therapeutic translation will depend on matching senolytics, senomorphics, immune-recalibrating approaches, microbiota-derived metabolites, and bone-targeted delivery systems to disease stage, dominant cell population, and measurable skeletal endpoints.

## 1 Introduction

Aging-related bone diseases are still appropriately grounded in the classical framework of uncoupled bone formation and bone resorption. The limitation of that framework is not that it is wrong, but that it does not fully explain chronic marrow inflammation, immune-cell remodeling, loss of regenerative capacity, deterioration of the vascular and stromal niche, and imaging-detectable microarchitectural damage in older individuals. Osteoimmunology provides the appropriate biological context because bone and immune systems share cytokines, stromal niches, hematopoietic environments, and osteoclastogenic pathways (1, 2). Recent primary studies further show that aging remodels skeletal stem/progenitor populations and osteoclast precursors (3–5). Aged marrow macrophages and endothelial niche cells also contribute to skeletal aging phenotypes (6, 7). These data support the view that skeletal aging is a multicellular tissue process rather than a simple osteoblast-osteoclast imbalance. Aging makes this relationship more consequential. Immune dysfunction and skeletal-cell stress develop together within bone marrow, joints, periodontal tissues, and fracture-repair niches, and their interaction shapes the phenotype of skeletal aging.

Cellular senescence offers one explanation for this convergence. Senescent cells enter durable cell-cycle arrest and acquire a senescence-associated secretory phenotype capable of changing neighboring cells through cytokines, chemokines, proteases, growth factors, and extracellular vesicles (8–10). In bone, senescent osteocytes, osteoblast-lineage cells, bone marrow stromal cells, and immune-related cells should be interpreted with attention to the model system and marker panel used. Mouse studies provide strong causal support that senescent-cell targeting can prevent age-related bone loss, whereas human evidence is strongest for associations in tissues and for biomarker-stratified clinical responses rather than for direct demonstration of senescent-cell clearance within bone (11–13). This distinction is important because evidence derived from genetic mouse models, ovariectomy, radiation, fracture repair, or human blood biomarkers does not carry the same inferential weight.

Immune remodeling is therefore best viewed as a mechanistic modifier of skeletal aging rather than a process that simply follows bone loss. Primary studies show that Th17 cells and CD4^+^CD28^−^ T cells in rheumatoid arthritis can influence osteoclastogenesis in specific experimental settings (14, 15). B-cell regulation of the RANKL/OPG axis, macrophage-derived grancalcin, and NK-cell surveillance pathways further show how immune compartments modify bone remodeling or senescent-cell clearance (16–18). Osteocyte-derived RANKL is required for age-associated cortical bone loss and is induced by senescence in mice, linking local cellular aging to bone resorption in that model (19). These findings support an osteoimmune view of skeletal aging, but they should not be read as proof that every immune-aging feature is causal in every human bone disease.

The same logic extends beyond the local marrow niche. Sex steroid deficiency-associated bone loss is microbiota-dependent and preventable by probiotics in mice, indicating that intestinal signals can tune inflammatory bone loss (20). Short-chain fatty acids provide a clearer mechanistic example: they are microbial fermentation products of dietary fiber that can act on host immune regulation and directly suppress osteoclast metabolic programs in experimental bone loss models (21–23). Thus, microbial metabolism enters the osteoimmune circuit because gut-derived metabolites can modify immune-cell balance, osteoclast precursor function, and systemic inflammatory tone rather than because taxonomic dysbiosis alone proves causality. These findings do not mean that every microbiota signature is causal, but they show that the gut, immune system, and skeleton cannot be considered in isolation.

Imaging gives this framework a translational anchor. Human skeletal tissue is difficult to sample repeatedly, and senescent-cell burden is not measurable in routine practice. DXA remains the clinical entry point for osteoporosis assessment (24). HR-pQCT resolves cortical and trabecular microarchitecture *in vivo* (25). MRI and MR spectroscopy characterize marrow adiposity or inflammatory marrow lesions (26, 27). 18F-NaF PET/CT quantifies regional bone turnover (28), and CBCT defines localized alveolar defects in selected periodontal settings (29). These modalities do not diagnose senescence, but they connect molecular hypotheses with structural damage, progression, and response to therapy.

**Figure 1:**
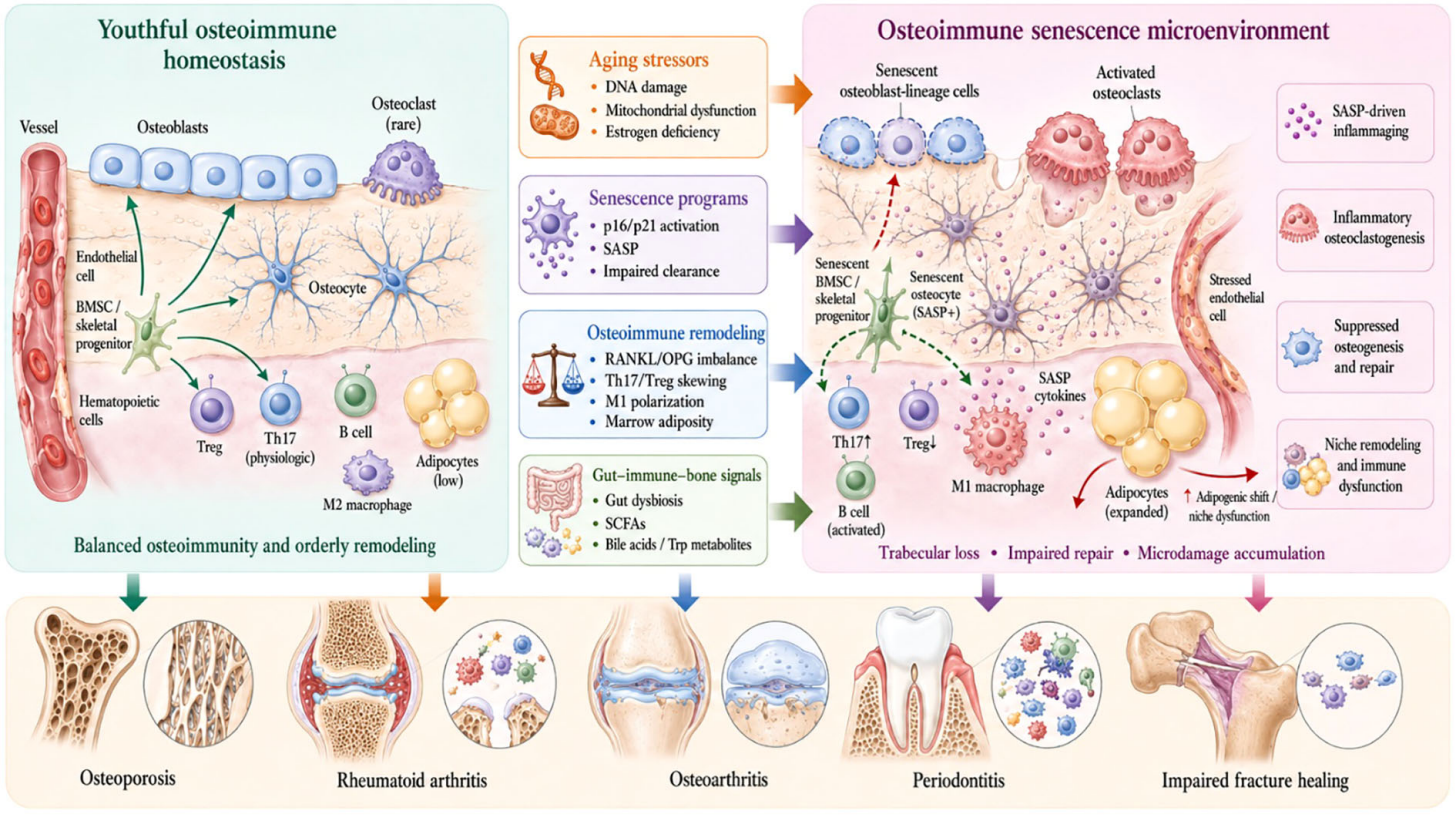
Osteoimmune senescence as an integrative framework linking aging stressors to skeletal degeneration. The figure contrasts youthful osteoimmune homeostasis with the senescent bone microenvironment. Aging-related DNA damage, mitochondrial dysfunction, estrogen deficiency, gut-derived inflammatory signals, and impaired senescent-cell clearance activate p16/p21 signaling and SASP production. These changes disturb RANKL/OPG balance, Th17/Treg regulation, macrophage polarization, marrow adiposity, endothelial support, and osteoblast-osteoclast coupling, leading to trabecular loss, microdamage accumulation, impaired repair, and fragility. The label referring to osteoblast-lineage dysfunction should be understood as senescent or less osteogenic osteoblast-lineage cells and related BMSCs/SSPCs, not as a normal terminal osteoblast fate. The framework links shared mechanisms across osteoporosis, rheumatoid arthritis-associated erosion, osteoarthritis, periodontitis-related alveolar bone loss, and delayed fracture healing. Mechanistic components are supported by representative primary studies and clinical data on senescent-cell targeting, osteocyte RANKL, and gut-derived metabolites (11, 19, 21). Additional evidence supports p21-positive fracture-repair cells, aged macrophage effects, and endothelial niche aging (6, 17, 34). OPG, osteoprotegerin; RANKL, receptor activator of nuclear factor-κB ligand; SASP, senescence-associated secretory phenotype; SCFAs, short-chain fatty acids; Trp, tryptophan.

We use osteoimmune senescence as a unifying but not generic concept. It emphasizes the convergence of senescent skeletal cells, aging immune compartments, SASP-driven inflammation, impaired senescent-cell clearance, gut-derived metabolic signals, imaging-detectable deterioration, and defective repair. By applying this concept to osteoporosis, rheumatoid arthritis-associated bone erosion, osteoarthritis, periodontitis-related alveolar bone loss, and impaired fracture healing, this review identifies shared mechanisms while preserving disease-specific endotypes. **Figure 1** summarizes how aging stressors convert osteoimmune homeostasis into SASP-driven inflammation, immune dysfunction, excessive osteoclastogenesis, impaired osteogenesis, and skeletal degeneration.

## 2 Conceptualizing osteoimmune senescence

Osteoimmune senescence denotes an aging-associated disease state in which senescent skeletal cells, remodeled immune compartments, impaired immune surveillance, chronic SASP signaling, and altered marrow-niche cues converge to disrupt skeletal homeostasis. The term is useful because it shifts age-related bone disease from a two-cell osteoblast-osteoclast model toward a tissue-level disorder involving cellular stress, immune remodeling, inflammaging, and failed repair. Aging has been linked to altered osteoimmune function through marrow niche deterioration, immune-cell dysfunction, inflammatory activation, and loss of bone homeostasis (30). Cellular senescence in bone is likewise recognized as a disease-modifying process rather than a passive marker of chronological age (8).

### 2.1 Definition and conceptual boundaries

Cellular senescence is now understood as a dynamic stress-response program characterized by stable proliferative arrest, apoptosis resistance, altered metabolism, chromatin and DNA-damage responses, and a context-dependent senescence-associated secretory phenotype rather than by any single marker (9, 31, 32). In aging bone, senescent cells may include osteocytes, osteoblast-lineage cells, bone marrow stromal cells, and immune-related cells, but classification should specify the marker set used, such as SA-β-gal activity, p16INK4a, p21CIP1, γH2AX/53BP1, telomere-associated DNA-damage foci, SASP factors, or functional resistance to proliferation (12, 33, 34). Immunosenescence is related but not identical to cellular senescence. It refers to broader age-related immune remodeling, including reduced naïve lymphocyte pools, expansion of differentiated memory cells, myeloid skewing, impaired surveillance, and weaker resolution responses. Exhausted immune cells are also distinct: they usually arise after chronic antigen stimulation and are characterized by impaired effector function and checkpoint-marker expression, whereas many so-called immunosenescent T cells remain highly inflammatory and active (35).

We use osteoimmune senescence to describe the interface at which senescent bone-lineage cells and aging immune compartments jointly disturb remodeling, repair, and inflammatory resolution. It is not a synonym for osteoporosis, although osteoporosis is the clearest systemic manifestation. The same biology is relevant to rheumatoid arthritis-associated bone erosion, osteoarthritis-related subchondral remodeling, periodontitis-associated alveolar bone loss, and impaired fracture healing. Its conceptual basis lies in osteoimmunology, which recognizes shared cellular niches and regulatory pathways between skeletal and immune systems (1, 36). The causal relevance of this interface is supported by evidence that targeting senescent cells prevents age-related bone loss in mice (11).

### 2.2 A three-layer framework

A practical way to organize osteoimmune senescence is to separate it into three interacting layers. The first is the skeletal-cell layer, which includes osteocytes, osteoblast-lineage cells, bone marrow mesenchymal stromal cells, skeletal stem/progenitor cells, and osteoclast precursors. Osteocytes regulate remodeling through RANKL/OPG and sclerostin-Wnt-related pathways (37–39). Mouse genetic studies identify osteocyte RANKL as a key driver of age-associated cortical bone loss (19). Recent lineage and single-cell studies also emphasize that BMSCs/SSPCs are heterogeneous: Fgfr3^+^ endosteal stem cells, Lepr^+^ stromal cells, and Z24-regulated SSPC populations have distinct roles in osteogenesis, mechanosensation, and age-related bone loss (3, 4, 40). The second layer is the immune compartment. T cells and B cells influence bone remodeling through cytokine production and RANKL or OPG regulation (15, 16). Macrophages/osteomacs and NK cells further contribute to repair regulation, efferocytosis, and surveillance of damaged or senescent cells (17, 18). Senescent CD4^+^CD28^−^ T cells promote osteoclastogenesis in rheumatoid arthritis, showing that immune aging can directly affect bone destruction in a chronic inflammatory setting (14).

**Figure 2:**
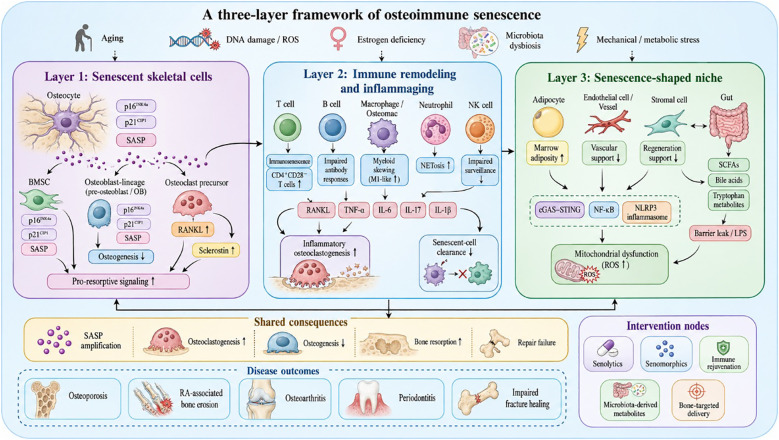
A three-layer framework of osteoimmune senescence in aging-related bone diseases. The first layer contains senescent skeletal cells, including osteocytes, BMSCs, osteoblast-lineage cells, SSPCs, and osteoclast precursors. The second layer contains remodeled immune compartments, including T cells, B cells, macrophages/osteomacs, neutrophils, and NK cells, which regulate inflammatory osteoclastogenesis and senescent-cell clearance. The third layer is the aging marrow niche, characterized by adiposity, vascular dysfunction, mitochondrial stress, cGAS-STING, NF-κB and NLRP3 activation, and gut-derived metabolic inputs. Together, these layers form a feed-forward loop that promotes SASP amplification, excessive resorption, weak formation, and defective repair. Representative evidence includes osteocyte RANKL, stromal or SSPC aging, and aged macrophage effects (17, 19, 42). Microbiota-dependent bone loss, SCFAs, and endothelial niche aging provide systemic and vascular support (6, 20, 21). BMSC, bone marrow mesenchymal stromal cell; SSPC, skeletal stem/progenitor cell; SASP, senescence-associated secretory phenotype; RANKL, receptor activator of nuclear factor-κB ligand; SCFAs, short-chain fatty acids; LPS, lipopolysaccharide; RA, rheumatoid arthritis.

The third layer is the aging marrow niche. Increased adiposity, reduced osteogenic potential, altered vascular support, myeloid bias, and persistent inflammatory signaling create a setting in which senescent cells influence each other rather than acting alone. This spatial view matters: bone aging is not a single-cell event but a niche-level process involving stromal cells, immune cells, endothelial cells, adipocytes, extracellular matrix, and microbial-metabolic signals (41, 42). **Figure 2** presents this three-layer model and shows how skeletal senescence, immune remodeling, and marrow-niche dysfunction form a feed-forward loop that favors bone resorption and defective repair.

### 2.3 Operational criteria and marker limitations

A persistent difficulty is the lack of a single reliable senescence or inflammaging marker. SA-β-gal activity, p16^INK4a^, p21^CIP1^, p53 activation, γH2AX/53BP1, telomere-associated DNA-damage foci, SASP factors, and inflammatory cytokines are informative, but each can also appear in stress, activation, differentiation, infection, trauma, or tissue injury responses (9, 10, 32). This ambiguity is especially important in bone, where osteocytes, osteoblasts, stromal cells, immune cells, and endothelial cells share overlapping stress programs (8, 12). Inflammaging is even harder to operationalize than cellular senescence because it is a tissue- and organism-level concept usually inferred from composite cytokine, innate immune, metabolic, microbial, and clinical frailty readouts rather than from one definitive assay. The distinction between p16-positive and p21-positive cells illustrates the problem. Targeted clearance of p21-positive cells, but not p16-positive cells, prevented radiation-induced osteoporosis and marrow adiposity in one model (33). In fracture repair, clearance of p21-positive cells accelerated healing, whereas p16-positive cell removal did not produce the same effect (34). Senescence programs therefore need to be defined by marker combinations, cell identity, tissue location, disease stage, and functional effect, rather than by one marker alone. **Table 1** summarizes practical marker categories and their limitations.

**Table 1:** Practical markers for cellular senescence and inflammaging in aging-related bone diseases.

Domain/readout	Representative markers or assays	What the readout indicates	Main limitation in bone and immune tissues
Cell-cycle arrest (9, 33, 34)	p16^INK4a^, p21^CIP1^, p53/Rb pathway activation, Ki-67 loss	Stable growth arrest and stress-response activation	Can also reflect transient stress, differentiation, activation, or injury; p16 and p21 programs are not interchangeable
DNA damage and telomere stress (9, 59, 60)	γH2AX, 53BP1, telomere-associated DNA-damage foci, micronuclei	Genotoxic stress that may initiate senescence and SASP	Also occurs during repair, oxidative injury, radiation exposure, and inflammation
Lysosomal and metabolic senescence (9, 32, 43)	SA-β-gal activity, GLB1, lipofuscin, mitochondrial dysfunction, ROS, NAD+/AMPK/mTOR changes	Senescence-associated metabolic and lysosomal remodeling	SA-β-gal is pH- and context-dependent; metabolic markers are not specific to irreversible senescence
SASP and inflammaging (10, 56, 116)	IL-6, IL-1β, TNF-α, CCL2, CXCLs, MMPs, HMGB1, PAI-1, circulating cytokine panels	Paracrine inflammatory output and systemic low-grade inflammation	Overlaps with infection, autoimmunity, obesity, trauma, menopause, and mechanical stress; source cells are often unknown
Immune aging and exhaustion (18, 35, 104)	CD28/CD27 loss, CD57, KLRG1, PD-1, TIM-3, LAG-3, NK cytotoxicity, efferocytosis assays	Age-related immune remodeling, surveillance capacity, or chronic antigen-driven exhaustion	Immunosenescence and exhaustion are not equivalent to classical cellular senescence; many immune cells remain proliferative or inflammatory
Bone-lineage functional readouts (11, 19, 37)	RANKL/OPG, sclerostin, ALP, osteocalcin, mineralization, osteoclastogenesis assays, bone turnover markers	Functional consequences of senescence or inflammaging on remodeling	These are skeletal effects rather than senescence-specific markers
Recommended composite strategy (13, 25, 33)	Marker panel + cell identity + spatial location + functional perturbation + imaging endpoint	Most robust approach for defining disease-driving osteoimmune senescence	Requires prospective validation and standardization across diseases

### 2.4 Why the concept matters

The value of this concept is explanatory and translational. It explains why osteoporosis, osteoarthritis, rheumatoid arthritis-associated erosion, periodontitis, and delayed fracture healing share chronic inflammation, altered immune-cell function, dysregulated osteoclastogenesis, impaired osteogenesis, and weak repair resolution despite their different triggers. It also clarifies why antiresorptive or anabolic drugs may not fully correct the underlying biology in every patient. Senolytics, senomorphics, immune-recalibrating approaches, microbiota-derived metabolites, and targeted delivery systems are therefore best viewed as complementary tools, not replacements for established skeletal therapies. At the same time, senescent-cell elimination must be selective and timed carefully, because transient senescence can support repair, immune recruitment, and tissue remodeling (8).

## 3 Cellular sources of osteoimmune senescence in aging bone

**Figure 3:**
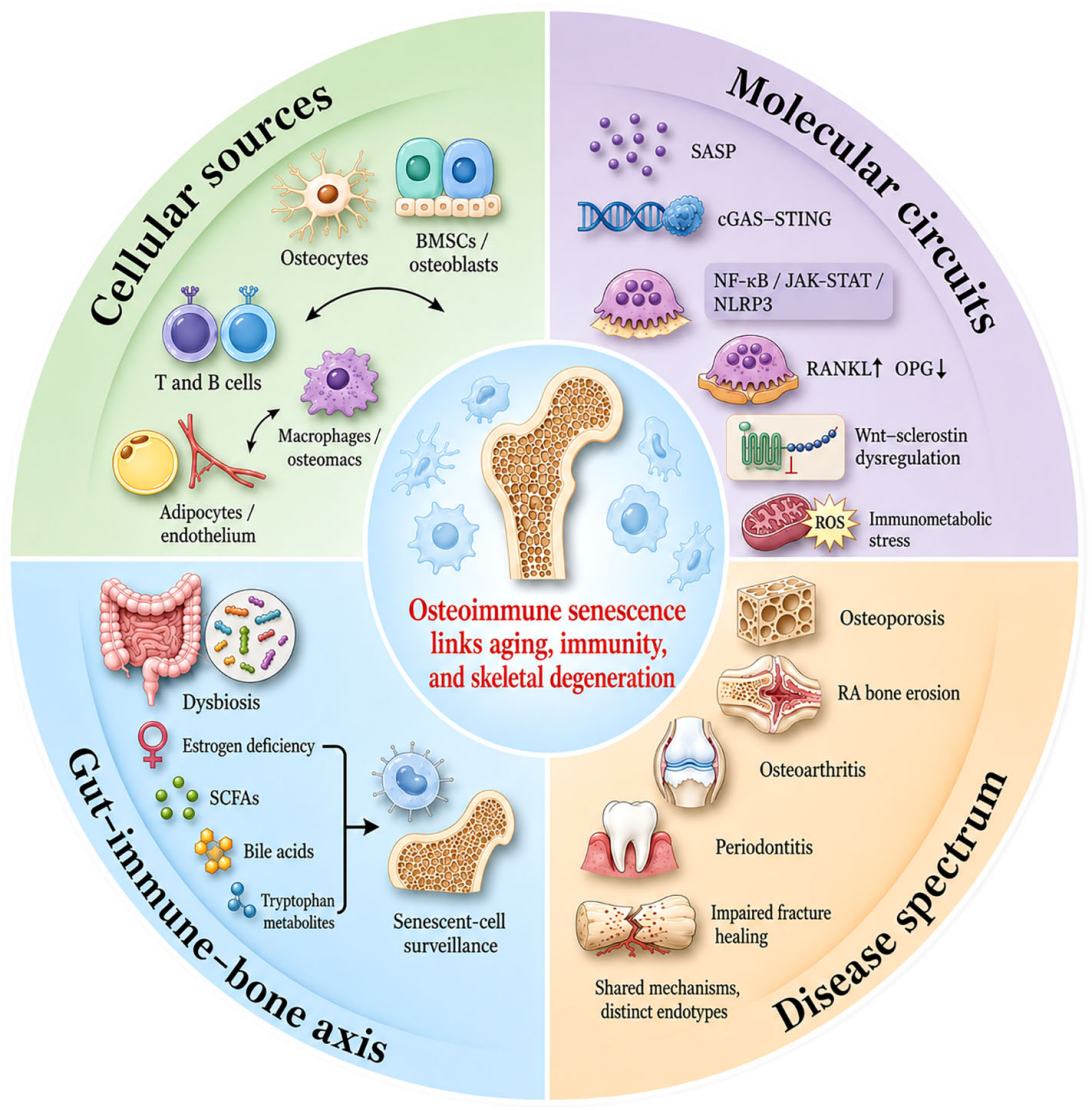
Cellular and molecular sources of osteoimmune senescence in aging-related bone diseases. Senescent osteocytes, BMSCs/osteoblast-lineage cells, T-cell aging/exhaustion, B-cell aging, aged macrophages/osteomacs, adipocytes, and endothelial cells shape the aging skeletal niche. Their interactions involve SASP signaling, cGAS-STING activation, NF-κB/JAK-STAT/NLRP3 pathways, RANKL/OPG imbalance, Wnt-sclerostin dysregulation, oxidative stress, and immunometabolic remodeling. The gut-immune-bone axis adds a systemic layer through dysbiosis, estrogen deficiency, SCFAs, bile acids, tryptophan metabolites, and impaired senescent-cell surveillance. These mechanisms converge in osteoporosis, rheumatoid arthritis-associated erosion, osteoarthritis, periodontitis, and impaired fracture healing while preserving disease-specific endotypes. The figure is supported by representative studies of senescent-cell targeting, osteocyte RANKL, and osteoblast-lineage senescence (11, 19, 43). Other evidence addresses macrophage-induced progenitor senescence, STING signaling in osteoarthritis, and gut-derived bile acid metabolites (17, 61, 77). Recent work on osteoclast precursor reprogramming, endothelial niche aging, and leptin-Lepr-driven SSC senescence illustrates disease-specific extensions (5, 6, 79). BMSCs, bone marrow mesenchymal stromal cells; SASP, senescence-associated secretory phenotype; SCFAs, short-chain fatty acids; RA, rheumatoid arthritis; RANKL, receptor activator of nuclear factor-κB ligand; OPG, osteoprotegerin.

Osteoimmune senescence does not arise from one pathogenic cell type. Evidence reviewed below identifies senescent osteocytes, senescent or dysfunctional osteoblast-lineage cells, aging BMSCs/SSPCs, inflammatory and menopause-reprogrammed osteoclast precursors, remodeled lymphocytes, aged macrophages, p21-positive neutrophil subsets, impaired NK-cell surveillance, marrow adipocytes, and endothelial cells as interacting contributors. Osteocyte, osteoblast-lineage, and SSPC data provide skeletal-cell support for this multicellular view (4, 19, 43). Immune-cell and endothelial-niche studies further support disease- and repair-specific contributions (6, 17, 34). Their relative contribution differs by disease, anatomical site, sex, endocrine state, microbial exposure, mechanical loading, and repair stage. This heterogeneity is central to the argument of this review: the same marker, such as p16INK4a or p21CIP1, can carry different biological meaning in osteoporosis, rheumatoid arthritis, osteoarthritis, periodontitis, and fracture repair. Therefore, each cell population below is discussed with attention to evidence strength, senescence markers, and the distinction between direct experimental causality and plausible extrapolation. **Figure 3** places these cellular sources within the molecular, microbial-metabolic, and disease-specific contexts discussed below.

### 3.1 Senescent osteocytes as organizers of skeletal inflammaging

Osteocytes are central to osteoimmune senescence because they are abundant, long-lived, and positioned within mineralized bone to coordinate remodeling. Osteocytes regulate osteoclastogenesis through RANKL/OPG-related signals and osteoblast activity through sclerostin-Wnt and mechanosensitive pathways (37–39). Osteocyte senescence has been characterized in experimental systems by combinations of p16INK4a, p21CIP1, DNA-damage markers, SA-β-gal activity, SASP factors, and functional changes in paracrine signaling (12, 44). In mice, osteocyte-derived RANKL is required for age-associated cortical bone loss and is induced by senescence, directly linking osteocyte aging to osteoclast-mediated bone resorption in that model (19). Osteocytes may also regulate the senescence status of other bone and marrow cells; radiation-induced osteocyte senescence altered BMSC differentiation potential through paracrine signaling (45). These data support the idea that senescent osteocytes can reshape the skeletal niche, but the degree to which this mechanism explains human osteoporosis still requires spatial validation in human bone. Osteocyte senescence may arise secondary to estrogen deficiency, oxidative stress, mechanical unloading, diabetes, glucocorticoid exposure, radiation, or accumulated microdamage. Future work needs to determine whether senescent osteocytes initiate bone loss, amplify existing skeletal damage, or mark a failed compensatory response.

### 3.2 BMSCs and osteoblast-lineage exhaustion

Bone marrow mesenchymal stromal cells and skeletal stem/progenitor cells are major contributors to osteoimmune senescence because they generate osteoblast-lineage cells and support the hematopoietic niche. With aging, BMSCs/SSPCs show reduced self-renewal, impaired osteogenic differentiation, increased adipogenic bias, mitochondrial dysfunction, DNA-damage responses, decreased mechanosensation, and expression of senescence-associated regulators (4, 41, 46). Recent work indicates that the aging skeletal progenitor compartment is heterogeneous. Fgfr3^+^ endosteal stem cells contribute to active osteogenesis in young bone marrow, whereas Lepr^+^ stromal cells become major osteoblast and adipocyte sources in adult marrow (3, 40). Premature aging of Z24-deficient SSPCs caused bone loss with reduced mechanosensation, and physical exercise partly reversed apoptosis, extracellular matrix defects, and bone mass loss in that model (4). Muscle-derived extracellular vesicles carrying miR-34a increase with age and can induce BMSC senescence, linking sarcopenia-like inter-organ communication to skeletal progenitor dysfunction (47). Senescent BMSCs are therefore not merely failed osteoblast precursors; their secretory phenotype can influence macrophage activation, osteoclast precursor survival, osteoblast differentiation, and immune-cell positioning (48). A limitation is that many BMSC senescence studies rely on *in vitro* expansion, oxidative stress, or replicative senescence models. *In vivo* BMSC aging is shaped by vascular supply, endocrine status, immune inflammation, mechanical loading, adiposity, and microbial-metabolic signals.

### 3.3 Osteoclast-lineage cells and inflammatory priming

The relationship between osteoclast-lineage cells and senescence requires caution. Mature osteoclasts are terminally differentiated multinucleated cells, and increased osteoclast activity in aging bone does not necessarily mean that mature osteoclasts undergo classical senescence. The stronger evidence supports inflammatory priming and intrinsic reprogramming of osteoclast precursors within a senescence-rich microenvironment. Senescent cell-conditioned medium can increase osteoclast progenitor survival and impair osteoblast mineralization, showing that SASP-like signals can shift remodeling toward bone resorption (11). Inflammatory cytokines such as TNF-α, IL-1β, IL-6, and IL-17 can promote osteoclastogenesis by increasing RANKL expression or sensitizing precursors to RANKL signaling (15, 49). Importantly, human and experimental data indicate that aging and menopause can reprogram osteoclast precursors toward more aggressive bone resorption, providing a mechanistic bridge between endocrine aging and inflammatory osteoclastogenesis (5). Current evidence therefore supports viewing osteoclast precursors as executors and amplifiers of osteoimmune senescence, while mature osteoclast senescence itself remains less clearly established. Therapeutically, targeting senescent niche cells, SASP pathways, RANKL signaling, or precursor reprogramming may be more rational than broadly labeling osteoclasts as senescent cells.

### 3.4 Senescent T cells as osteoclastogenic amplifiers

T cells provide a direct link between adaptive immune aging and inflammatory bone destruction, but terminology requires precision. Aging and chronic inflammation can reduce naïve T-cell diversity, expand differentiated memory and effector subsets, promote loss of CD28 or CD27, increase KLRG1 or CD57 expression, and alter cytokine production (14, 35). These cells are often called immunosenescent T cells, yet they are not always classically senescent because some remain metabolically active, cytotoxic, or pro-inflammatory. Exhausted T cells differ again because chronic antigen stimulation can drive checkpoint-marker expression and impaired effector function rather than a canonical SASP phenotype (35). In rheumatoid arthritis, CD4^+^CD28^−^ T cells with senescence-like features promote osteoclastogenesis more effectively than CD28^+^ T cells (14). Th17 cells also function as osteoclastogenic helper T cells by linking IL-17 production with RANKL-dependent bone resorption (15, 50). The contribution of senescent or senescence-like T cells may be strongest in inflammatory arthritis. Evidence from RA should not be overextended to all forms of age-related bone loss. In primary osteoporosis, T-cell aging may contribute to inflammaging and RANKL production, but direct causal evidence remains less complete than in RA.

### 3.5 B cells and the RANKL/OPG balance

B cells influence bone remodeling through both immune and skeletal mechanisms. They can produce RANKL and OPG, thereby regulating osteoclast differentiation depending on their activation state and disease context (16, 51, 52). B-cell control of the RANKL/OPG axis is a key example of osteoimmune regulation. In osteoimmune senescence, aging-related changes in B-cell development, repertoire diversity, and marrow niche interactions may disturb this balance. However, direct evidence that a clearly defined senescent B-cell subset accumulates in aging bone and causally drives skeletal deterioration remains limited. B cells should therefore be framed as plausible osteoimmune contributors rather than established primary senescent drivers. Their importance may be greater in autoimmune or infection-related bone loss than in uncomplicated skeletal aging. Future work needs to define B-cell aging states with single-cell phenotyping, RANKL/OPG profiling, markers of cellular senescence or exhaustion, and functional osteoclastogenesis assays.

### 3.6 Macrophages, osteomacs, and inflammatory niche remodeling

Macrophages are essential regulators of bone remodeling, efferocytosis, inflammatory resolution, angiogenesis, and fracture repair. Bone-associated macrophages, or osteomacs, support osteoblast function and bone formation under physiological conditions, whereas aging can shift macrophage states toward persistent inflammatory activation, impaired efferocytosis, and reduced repair competence (7, 17, 53). Aged macrophages can directly impair skeletal regeneration. Age-related secretion of grancalcin by macrophages promotes skeletal stem/progenitor cell senescence during fracture healing (17). Aged bone marrow macrophages can also propagate paracrine senescence through extracellular vesicles in mice, supporting the broader concept that aged myeloid cells can impose senescence-like dysfunction on neighboring tissues (7). The traditional M1/M2 macrophage model is inadequate for aging bone. Macrophages in osteoimmune senescence may show mixed inflammatory, senescence-associated, metabolic, and defective-resolution states. Single-cell, spatial, and metabolic approaches are needed to identify which macrophage states are pathogenic and which are required for repair.

### 3.7 Neutrophils and NK-cell surveillance

Neutrophils are increasingly recognized as active regulators of fracture healing and osteoimmune repair. p21-positive osteochondroprogenitor cells and neutrophil subsets expressing senescence-associated features have been identified in fracture callus. Targeted clearance of p21-positive cells suppressed senescence signatures and accelerated fracture healing (34). This evidence challenges the idea that all senescent cells have identical effects. p21-positive cells may impair repair in specific fracture contexts, while p16-positive cells may not have the same effect. NK cells add another layer because they participate in immune surveillance of senescent cells. Granule exocytosis and NKG2D ligand recognition can mediate immune surveillance of senescent cells in experimental models (18, 54). Direct evidence connecting NK-cell dysfunction to senescent-cell accumulation in bone is still limited, so NK-cell surveillance should be presented as a plausible mechanism requiring skeletal validation rather than as an established driver of bone loss.

### 3.8 Niche cells: adipocytes and endothelial cells

Compared with osteocytes, BMSCs, and immune cells, marrow adipocytes and endothelial cells are less firmly established as direct senescence drivers, but their niche-level effects are increasingly supported. Aging marrow shows increased adiposity, reduced osteogenic potential, altered vascular support, and chronic inflammatory signaling (55). Marrow adipocytes can modify immune metabolism, hematopoiesis, and inflammatory tone. Endothelial aging can compromise angiogenesis, immune-cell trafficking, nutrient delivery, and progenitor support. A recent primary study showed that endothelial BMAL1 decline during aging led to bone loss in mice by destabilizing extracellular fibrillin-1 and activating TGF-β/SMAD3 signaling, thereby linking endothelial niche aging to BMSC depletion and osteoclast formation (6). These data strengthen the rationale for including endothelial cells in the osteoimmune senescence niche, while also showing that the mechanism may be endothelial dysfunction rather than classical cellular senescence alone.

## 4 Molecular circuits linking senescence, immunity, and bone remodeling

**Figure 4:**
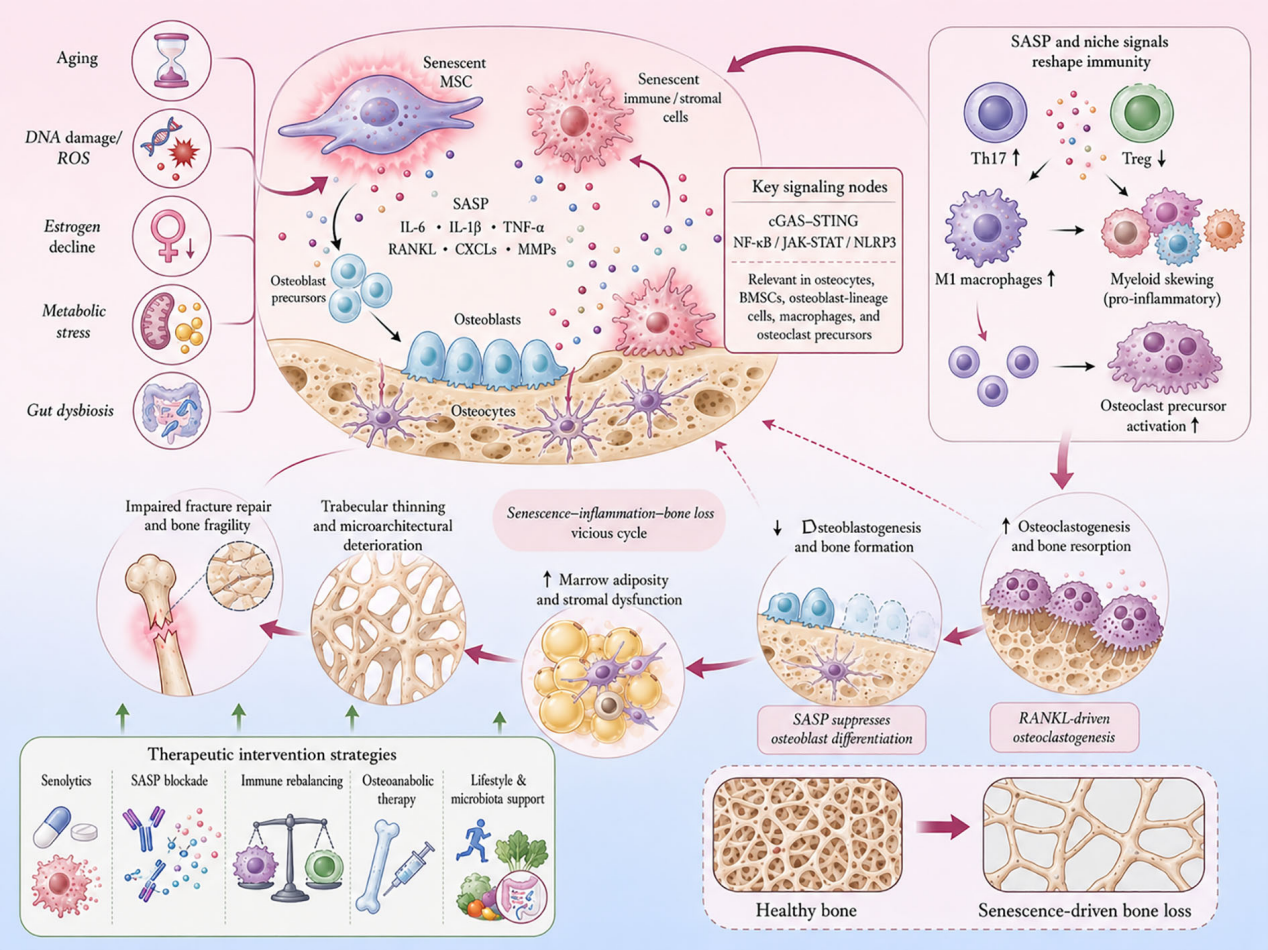
Senescence-inflammation-bone loss vicious cycle in aging-related osteoimmune remodeling. Aging-related DNA damage, reactive oxygen species, estrogen decline, metabolic stress, and gut dysbiosis induce cellular senescence in the bone marrow microenvironment. Senescent stromal, immune, and osteoblast-lineage cells release SASP mediators, including IL-6, IL-1β, TNF-α, RANKL, CXCL chemokines, and MMPs. These signals suppress osteoblast differentiation, increase osteoclast precursor activation, and reinforce Th17 polarization, reduced Treg control, M1-like macrophage activation, and myeloid skewing. The resulting loop promotes marrow adiposity, stromal dysfunction, microarchitectural deterioration, impaired fracture repair, and bone fragility. Representative evidence includes senescent-cell targeting in bone, osteocyte RANKL, and SCFA effects on osteoclast metabolism (11, 19, 21). Other studies support Th17-driven osteoclastogenesis, macrophage-induced progenitor senescence, and STING/NF-κB-related osteoarthritis signaling (15, 17, 61). SASP and NLRP3-targeted modulation support inflammatory control (64, 65). cGAS-mediated senescence signaling and osteoclast precursor reprogramming provide additional mechanistic support (5, 59). CXCLs, C-X-C motif chemokine ligands; IL, interleukin; MMPs, matrix metalloproteinases; MSC, mesenchymal stromal cell; RANKL, receptor activator of nuclear factor-κB ligand; ROS, reactive oxygen species; SASP, senescence-associated secretory phenotype; TNF-α, tumor necrosis factor-α; Treg, regulatory T cell.

At the molecular level, osteoimmune senescence is best understood as a feed-forward inflammatory circuit. DNA damage and mitochondrial stress initiate senescence programs; cGAS-STING, NF-κB, p38 MAPK, JAK/STAT, and NLRP3 pathways amplify SASP output; and RANKL/OPG or Wnt-sclerostin imbalance translates this inflammatory state into excessive resorption and impaired formation. These pathways are often discussed separately, but their pathological force comes from their interaction. **Figure 4** depicts this cycle, in which SASP mediators suppress osteoblast differentiation, activate osteoclastogenesis, and reinforce Th17 polarization, M1-like macrophage activation, marrow adiposity, and skeletal fragility.

### 4.1 SASP as the inflammatory language of osteoimmune senescence

The senescence-associated secretory phenotype is the major communication program through which senescent cells influence neighboring cells. SASP includes cytokines, chemokines, growth factors, proteases, extracellular matrix regulators, DAMPs, and extracellular vesicles. In bone, SASP-producing osteocytes, stromal cells, osteoblast-lineage cells, and immune cells can create a pro-resorptive and anti-regenerative microenvironment (8, 56). SASP can promote osteoclastogenesis by increasing inflammatory cytokines and chemokines that recruit or activate osteoclast precursors. It can also suppress osteoblast differentiation and impair matrix mineralization. Senescent cell-conditioned medium has been shown to impair osteoblast mineralization and enhance osteoclast progenitor survival (11). SASP is not a fixed list of inflammatory factors. Its composition depends on cell type, inducer, tissue environment, disease stage, and duration of senescence (57). Acute SASP may support immune recruitment and repair, whereas chronic SASP can maintain inflammation, matrix degradation, stem-cell exhaustion, and tissue dysfunction (8).

### 4.2 DNA damage, mitochondria, and cGAS–STING signaling

DNA damage responses are major upstream drivers of cellular senescence. Telomere attrition, oxidative stress, radiation, glucocorticoids, metabolic stress, and mechanical injury can activate p53/p21 or p16/Rb pathways. In skeletal tissues, these responses may occur in osteocytes, osteoblast-lineage cells, stromal cells, chondrocytes, endothelial cells, and immune cells (12, 58). Mitochondrial dysfunction connects senescence with inflammatory remodeling through impaired respiration, increased reactive oxygen species, defective mitophagy, and mitochondrial DNA leakage. The cGAS-STING pathway is important because it links cytosolic DNA sensing to innate immune activation. Cytoplasmic chromatin fragments activate cGAS-STING and promote SASP production in senescent cells, whereas cytosolic chromatin sensing through cGAS can reinforce cellular senescence (59, 60). Mitochondrial DNA may provide an additional source of cytosolic DNA under stress. In osteoarthritis, STING signaling has been linked to chondrocyte senescence, apoptosis, matrix degradation, and disease progression (61). The role of cGAS-STING in osteoimmune senescence is likely context-dependent. It may protect tissues by detecting damage or infection, but persistent activation can drive chronic inflammation. Future work needs to determine when cGAS-STING functions as a protective surveillance pathway and when it becomes a driver of skeletal inflammaging.

### 4.3 NF-κB, p38 MAPK, JAK/STAT, and NLRP3

NF-κB is a central transcriptional regulator of inflammatory SASP, but its cell-type context must be specified. In osteoblast-lineage cells and BMSCs, persistent NF-κB activation can suppress osteogenesis and promote inflammatory mediator release. In osteocytes and chondrocytes, it can couple DNA damage, oxidative stress, or cGAS-STING activation to SASP-like cytokine and matrix-degrading enzyme production. In chondrocytes and senescent cells, NF-κB can amplify STING- or senescence-associated inflammatory programs (61–63). Therefore, the same pathway may drive osteoblast suppression, cartilage catabolism, macrophage activation, or osteoclastogenesis depending on the cell type. p38 MAPK also contributes to senescence-associated inflammatory signaling. JAK/STAT signaling is another SASP-related pathway with therapeutic relevance because JAK inhibition reduces senescence-associated inflammatory mediators in aging models (64). The NLRP3 inflammasome links sterile inflammation, metabolic stress, and bone loss; osteoblast-specific NLRP3 down-regulation has been reported to protect against postmenopausal osteoporosis in experimental research (65).

### 4.4 RANKL/OPG imbalance and inflammatory osteoclastogenesis

The RANKL/RANK/OPG system is the core osteoimmune pathway controlling osteoclast differentiation (66, 67). RANKL promotes osteoclastogenesis, whereas OPG acts as a decoy receptor that limits RANKL activity. Because RANKL and OPG can be regulated by skeletal cells and immune cells, this axis directly connects immune inflammation with bone resorption. Senescent osteocytes are especially relevant to this pathway. Osteocyte-derived RANKL is required for cortical bone loss with age, and senescence can induce osteocyte RANKL expression (19, 68). This provides a direct mechanism by which osteocyte senescence can be translated into age-related bone resorption. Inflammatory cytokines can amplify RANKL-dependent osteoclastogenesis. IL-17 promotes osteoclastogenesis by linking T-cell activation to bone destruction, particularly in inflammatory arthritis (15). TNF-α and IL-1β can also cooperate with RANKL to enhance osteoclast precursor differentiation. RANKL/OPG imbalance is not a senescence-specific marker. Estrogen deficiency, glucocorticoids, disuse, autoimmune inflammation, infection, and metabolic disease can all alter this pathway. The senescence-specific contribution should be supported by evidence that senescent-cell burden or SASP mediators directly alter RANKL/OPG signaling.

### 4.5 Wnt/sclerostin dysregulation and impaired bone formation

Suppressed bone formation is a central feature of osteoimmune senescence. Wnt/β-catenin signaling promotes osteoblast differentiation, survival, and bone formation. Sclerostin, mainly produced by osteocytes, inhibits Wnt signaling and limits osteoblast activity (38, 39). Sclerostin has been linked to bone aging because aging is associated with reduced bone formation and altered osteocyte signaling. Sclerostin inhibits the Wnt pathway by interacting with LRP5/6 and thereby restrains bone formation (69). Senescent osteocytes may therefore impair bone formation through both SASP activity and anti-anabolic sclerostin-related signaling. Inflammatory cytokines may further suppress osteoblast differentiation and mineralization. Thus, impaired bone formation in osteoimmune senescence is likely caused by both cell-intrinsic osteoblast-lineage exhaustion and extrinsic inflammatory inhibition. Anti-sclerostin strategies may be useful, but their effects may depend on whether the inflammatory and senescent niche is also controlled.

### 4.6 Immunometabolic reprogramming

Immunometabolic remodeling links senescence, inflammation, and bone turnover. Senescent cells often show mitochondrial dysfunction, altered glycolysis, NAD+ decline, mTOR activation, AMPK suppression, defective autophagy, and lipid metabolic changes. These changes can affect osteoblast differentiation, osteoclast activity, marrow adiposity, and immune-cell function. Osteoclast differentiation requires metabolic adaptation. Short-chain fatty acids can suppress osteoclast metabolism and protect against pathological bone loss, indicating that metabolic regulation can directly control osteoclastogenesis (21, 22). This mechanism connects microbial metabolism, immune regulation, and bone resorption. Metabolic checkpoint failure can also promote osteoblast-lineage senescence. Men1 loss-induced osteoblast senescence involved mTORC1 activation and AMPK suppression, and metformin rescued aspects of this phenotype (43). This indicates that mTOR/AMPK imbalance may be a therapeutic node in osteoblast senescence. A limitation is that many immunometabolic studies rely on static metabolite levels. Metabolite abundance does not necessarily reflect pathway flux, tissue exposure, or cell-specific utilization. Future work needs to incorporate isotope tracing, single-cell metabolic profiling, mitochondrial assays, and spatial metabolomics.

## 5 Gut–immune–bone–senescence axis: an underexplored dimension

The gut-immune-bone axis adds a systemic layer to the senescence model. Aging alters microbial ecology, weakens barrier function, increases microbial-product exposure, changes metabolite production, and promotes low-grade inflammation. These changes reshape T-cell balance, macrophage activation, osteoclast precursor metabolism, osteoblast function, and marrow-niche homeostasis. The gut therefore acts less as a distant metabolic organ than as a modulator of inflammatory tone and skeletal immune remodeling.

### 5.1 Gut dysbiosis and skeletal inflammaging

The gut microbiota can regulate bone mass in mice, establishing an experimental foundation for the gut–bone axis (70, 71). Microbial colonization can also regulate bone formation and growth through IGF-1, indicating that the microbiota affects anabolic skeletal pathways as well as resorptive pathways (72). Aging-associated dysbiosis may promote skeletal inflammaging by increasing barrier permeability and microbial product exposure (73). Microbial products can activate innate immune pathways, including TLR4/NF-κB signaling, macrophage inflammatory priming, and osteoclast precursor sensitization. These signals may increase TNF-α, IL-6, IL-1β, IL-17, and RANKL-related signaling in bone-relevant tissues. This relationship is better interpreted critically. Gut microbial composition is shaped by diet, antibiotics, geography, sex hormones, frailty, medications, physical activity, and comorbidities. Therefore, dysbiosis is not a single causal entity. Accordingly, future work needs to integrate functional microbial signatures, barrier measures, immune mediators, metabolite profiles, and skeletal endpoints rather than relying on taxonomic dysbiosis alone (73, 74).

### 5.2 Estrogen deficiency as a gut–immune–bone trigger

Sex steroid deficiency provides one of the strongest examples of gut-immune-bone interaction. Sex steroid deficiency-associated bone loss is microbiota-dependent and can be prevented by probiotics in mice (20). Ovariectomy-induced bone loss has also been linked to microbial-dependent expansion of bone marrow Th17 cells and TNF-α-producing T cells (75). These findings do not imply that all postmenopausal women already have advanced inflammaging at the onset of menopause. Rather, estrogen deficiency may create an inflammatory and microbial-barrier shift that converges with incipient age-related inflammation, osteoclast precursor reprogramming, and marrow niche aging during midlife and later aging (5, 20, 75). Human studies suggest that postmenopausal osteoporosis is associated with altered gut microbial composition (76). These associations are important but remain vulnerable to confounding by diet, age, body mass index, medications, calcium intake, and regional background. Therefore, human microbiome signatures are best considered hypothesis-generating unless supported by longitudinal validation and mechanistic testing.

### 5.3 Short-chain fatty acids as senomorphic-like metabolites

Short-chain fatty acids are among the best-studied microbial metabolites in bone regulation. Acetate, propionate, and butyrate can influence intestinal barrier integrity, Treg differentiation, HDAC activity, G-protein-coupled receptor signaling, and inflammatory tone. SCFAs regulate systemic bone mass and protect from pathological bone loss by suppressing osteoclast metabolism (21, 22). Butyrate and propionate can also regulate colonic regulatory T-cell homeostasis, providing a link between microbial metabolism and immune tolerance (23). In an osteoimmune senescence framework, SCFAs may function as senomorphic-like metabolites because they may reduce inflammatory propagation without necessarily eliminating senescent cells. Their effects may include barrier restoration, Treg support, osteoclast metabolic suppression, and reduction of inflammatory osteoclastogenesis. SCFAs should not be described as universally beneficial. Their effects depend on dose, receptor expression, immune context, diet, microbial ecology, host age, and disease stage. Fecal SCFA levels also do not necessarily reflect production rate, absorption, systemic exposure, or bone marrow availability. Causal studies should combine metabolite quantification with immune phenotyping and bone histomorphometry.

### 5.4 Bile acids and tryptophan metabolites

Bile acids are modified by gut bacteria and can regulate metabolism and immunity through receptors such as FXR, TGR5, and constitutive androstane receptor-related pathways. Glycolithocholic acid increases circulating Treg frequency through constitutive androstane receptor signaling and alleviates postmenopausal osteoporosis (77). This provides a direct link among microbial bile acid metabolism, immune regulation, and bone protection. Tryptophan metabolites provide another emerging gut-derived osteoimmune signal. Microbial tryptophan metabolites can regulate intestinal AhR signaling, barrier function, and immune responses. Microbial tryptophan metabolites ameliorate ovariectomy-induced bone loss by repairing intestinal AhR-mediated gut–bone signaling (78). These pathways should be discussed with nuance. Bile acid and tryptophan metabolism are complex, and not all metabolites are protective. Some microbial metabolites may promote immune tolerance, whereas others may contribute to inflammation, metabolic stress, or impaired musculoskeletal stem-cell function. The key question is which metabolite acts through which receptor in which host context.

### 5.5 Endocrine-nutritional signals: leptin, ghrelin, diet, and lifestyle

Gut-derived metabolism also intersects with endocrine and lifestyle signals that may influence skeletal inflammaging. Leptin is especially relevant because LepR^+^ mesenchymal stromal cells are a major source of adult marrow osteoblasts and adipocytes (40). Recent single-cell work in osteoarthritis further suggests that leptin-Lepr signaling can promote senescence of Lepr^+^ skeletal stem cells through a STAT3-FGF7 axis, thereby worsening subchondral bone remodeling in experimental disease (79). This does not prove that leptin is a universal senescence driver in osteoporosis, but it supports testing leptin signaling as a context-dependent link between adiposity, inflammation, progenitor aging, and bone remodeling. Ghrelin and its receptor may also influence bone through appetite, growth hormone, inflammation, and osteoblast-lineage effects, but direct evidence connecting ghrelin signaling to cellular senescence in aging bone remains limited. Diet and lifestyle should therefore be discussed as modulators of inflammaging rather than as established senolytics. Dietary fiber can increase SCFA availability, and mechanical loading or exercise can improve progenitor mechanosensation and bone mass in aging-related models (4, 21).

### 5.6 Microbiota and senescent-cell surveillance

A distinctive but less proven aspect of the gut–immune–bone–senescence axis is senescent-cell surveillance. Senescent cells can be cleared by immune cells, including macrophages and NK cells. Aging-associated dysbiosis might impair this clearance by promoting chronic inflammatory noise while reducing effective immune resolution. Direct evidence that gut dysbiosis impairs clearance of senescent osteocytes or BMSCs remains limited. This mechanism is therefore best framed as an emerging hypothesis rather than an established pathway. Future work needs to combine microbiota manipulation with senescent-cell mapping, NK-cell and macrophage functional assays, SASP profiling, and skeletal phenotyping.

### 5.7 Therapeutic implications

Microbiota-targeted interventions may include probiotics, prebiotics, postbiotics, dietary fiber, fecal microbiota transplantation, and microbial metabolite supplementation. Probiotics prevented sex steroid deficiency-associated bone loss in mice, supporting the therapeutic potential of barrier restoration and immune modulation (20). Human evidence also suggests that Lactobacillus reuteri 6475 can reduce bone loss in older women with low BMD (80). SCFA enhancement may suppress osteoclast metabolism and reduce inflammatory bone loss (21). Bile acid- and tryptophan-derived metabolite strategies may provide more mechanism-specific approaches. GLCA-based regulation of Tregs and tryptophan metabolite-mediated AhR signaling suggest that microbial metabolites can be mapped to defined osteoimmune pathways (77). These approaches may be especially relevant to postmenopausal osteoporosis and inflammation-dominant skeletal aging. The translational limitation is that many microbiome interventions remain preclinical. Future trials need to integrate BMD, bone turnover markers, fracture risk, immune-cell phenotypes, gut permeability, microbial metabolites, and inflammatory mediators. Without multidimensional endpoints, it will be difficult to determine whether microbiome interventions truly modify osteoimmune senescence.

## 6 Disease spectrum of osteoimmune senescence

**Figure 5:**
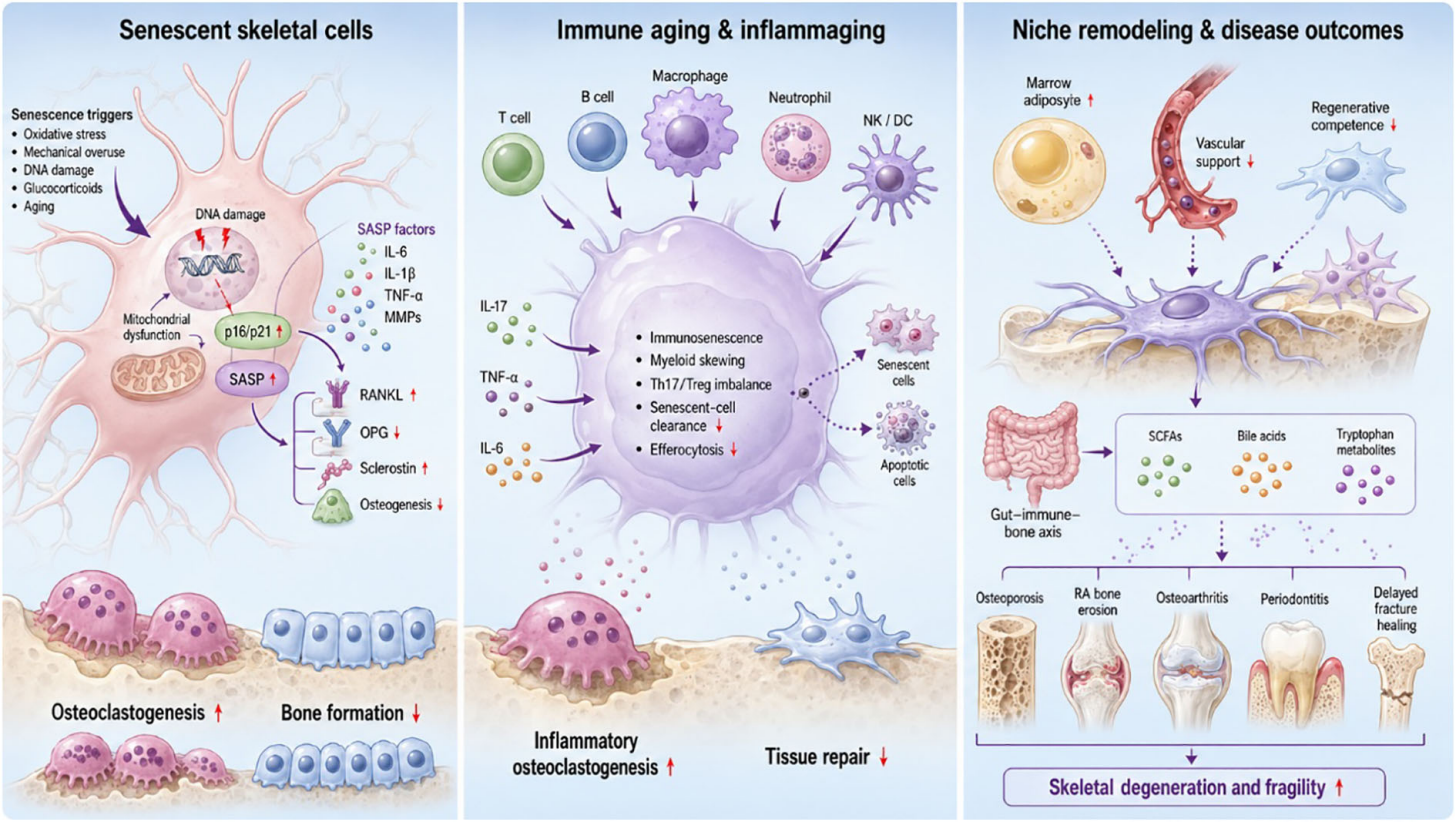
Osteoimmune senescence links senescent skeletal cells, immune aging, niche remodeling, and aging-related bone diseases. Senescent skeletal cells generate SASP factors that increase RANKL, reduce OPG signaling, induce sclerostin, enhance osteoclastogenesis, and suppress bone formation. Immune aging amplifies this process through myeloid skewing, Th17/Treg imbalance, impaired senescent-cell clearance, defective efferocytosis, and persistent inflammatory mediator production. Marrow adiposity, vascular decline, reduced regenerative competence, and gut-derived metabolic signals further remodel the skeletal niche. These interactions contribute to osteoporosis, rheumatoid arthritis-associated erosion, osteoarthritis, periodontitis-related alveolar bone loss, delayed fracture healing, and skeletal fragility. Disease links are supported by representative evidence from senescent-cell targeting in osteoporosis and OA, and senescent T cells in RA (11, 14, 81). p21-positive cells in fracture repair, periodontal osteocyte senescence, and macrophage-induced progenitor senescence provide additional disease-specific examples (17, 34, 83). The phase 2 D+Q trial and osteoclast precursor or Lepr+ SSC studies illustrate translational and endotype-specific extensions (5, 13, 79). SASP, senescence-associated secretory phenotype; RANKL, receptor activator of nuclear factor-κB ligand; OPG, osteoprotegerin; MMPs, matrix metalloproteinases; SCFAs, short-chain fatty acids; RA, rheumatoid arthritis.

Osteoimmune senescence provides a disease-spanning framework for aging-related skeletal disorders, but the strength of evidence differs markedly across diseases. Osteoporosis has the strongest causal evidence from mouse senescent-cell targeting and an emerging biomarker-stratified human senolytic trial (11, 13). Osteoarthritis and fracture repair have direct preclinical evidence for local senescent-cell targeting and p21-positive repair-cell populations (34, 81). Rheumatoid arthritis-associated erosion has strong evidence for immune-mediated osteoclastogenesis and senescence-like CD4^+^CD28^−^ T-cell effects, but autoimmune mechanisms remain primary (14, 15, 82). Periodontitis has evidence for microbial product-induced osteocyte senescence and local inflammation-accelerated senescence (83, 84). By contrast, B cells, NK cells, marrow adipocytes, and some endothelial or microbiota-related mechanisms remain more speculative in direct skeletal senescence terms. Thus, these diseases should not be forced into one uniform model. Instead, each may represent a distinct osteoimmune senescence endotype shaped by local tissue architecture, systemic aging, endocrine state, microbial exposure, mechanical stress, and immune-inflammatory context. **Figure 5** integrates senescent skeletal cells, immune aging, niche remodeling, and disease outcomes, while disease-specific imaging phenotypes anchor this framework to measurable clinical pathology.

### 6.1 Osteoporosis as the prototype

Osteoporosis is the most representative disease model for osteoimmune senescence. It combines age-related bone loss, impaired bone formation, increased bone resorption, marrow niche aging, immune dysregulation, and fracture susceptibility. Targeting senescent cells prevented age-related bone loss in mice, showing that senescent cells can actively drive skeletal deterioration (11, 85). Osteocytes are likely central mediators in osteoporotic senescence. Osteocyte senescence is linked to bone loss associated with aging and other skeletal stress states (44). Osteocyte-derived RANKL is required for age-related cortical bone loss and is induced by senescence (19). Postmenopausal osteoporosis adds an endocrine-immune layer. Estrogen deficiency can alter gut permeability, immune activation, Th17 expansion, cytokine production, and RANKL-related signaling (20). Thus, postmenopausal osteoporosis can be interpreted as an endocrine-triggered osteoimmune senescence phenotype. From an imaging perspective, osteoporosis should not be reduced to low BMD. HR-pQCT can provide compartment-specific measures of cortical thickness, cortical porosity, trabecular number, trabecular thickness, and estimated bone strength, whereas MRI-based marrow fat assessment may capture the adipogenic shift of the aging marrow niche (25, 26). 18F-NaF PET/CT may further complement structural imaging by assessing regional bone metabolic activity, which could be useful for monitoring early biological responses to senotherapeutic or anabolic interventions before large changes in BMD occur (28). Osteoporosis remains heterogeneous. Some patients may have senescent osteocyte-dominant disease, whereas others may have immune inflammation-dominant, gut dysbiosis-dominant, marrow adiposity-dominant, or low-anabolic endotypes. This heterogeneity may influence the response to senolytics, senomorphics, antiresorptives, and anabolic agents.

### 6.2 Rheumatoid arthritis-associated bone erosion

Rheumatoid arthritis-associated bone erosion is a strong model for the immune side of osteoimmune senescence. RA bone destruction occurs in a chronically inflamed synovial environment containing activated T cells, B cells, macrophages, dendritic cells, fibroblast-like synoviocytes, cytokines, and osteoclast precursors. Th17 cells link T-cell activation to osteoclastogenesis and bone destruction (15, 50). Senescent CD4^+^CD28^−^ T cells can promote osteoclastogenesis more strongly than CD28^+^ T cells in RA (14). This finding provides direct evidence that senescence-like immune remodeling can acquire bone-destructive function. It also suggests that immunosenescence may amplify inflammatory osteoclastogenesis in autoimmune bone loss. Fibroblast-like synoviocytes are also important because they translate immune inflammation into local tissue destruction. Synovial fibroblast-derived RANKL contributes to osteoclast formation and erosive disease in inflammatory arthritis (82). IL-17-associated PI3K/Akt and NF-kappaB signaling further illustrates how RA cytokine networks can amplify inflammatory synovial activation (86). Whether these fibroblasts are classically senescent or chronically activated remains an important unresolved question. RA should not be described as simply accelerated aging. Autoimmunity, citrullinated antigens, genetic susceptibility, stromal activation, and treatment exposure all shape RA bone erosion. Senescence is best interpreted as a disease-amplifying mechanism within chronic inflammatory osteoimmunology. Imaging is particularly informative in RA because bone damage evolves across synovium, cortical bone, and subchondral marrow. MRI-detected bone marrow edema or osteitis predicts erosive progression and has a histological correlate in inflammatory marrow infiltration adjacent to trabecular bone, supporting a direct link between immune inflammation and local bone destruction (27). Therefore, MRI-based osteitis, erosions, and synovitis may serve as tissue-level readouts of immune senescence-amplified osteoclastogenesis.

### 6.3 Osteoarthritis and subchondral bone remodeling

Osteoarthritis is a whole-joint disease involving cartilage, synovium, subchondral bone, meniscus, infrapatellar fat pad, immune cells, and sensory nerves. Cellular senescence has been implicated in age-associated OA and post-traumatic OA through chondrocyte dysfunction, SASP production, matrix-degrading enzymes, and inflammatory mediators (87, 88). Local clearance of senescent cells attenuated post-traumatic OA and created a pro-regenerative environment in experimental models (81). This provides strong evidence that senescent cells can actively contribute to joint degeneration rather than simply mark damaged cartilage (89). Subchondral bone is essential for integrating OA into an osteoimmune senescence framework. Abnormal subchondral bone remodeling can alter biomechanics, promote osteophyte formation, and interact with cartilage degeneration. Senescence-associated inflammation in cartilage and synovium may influence subchondral osteoblasts, osteoclasts, and marrow immune cells. MRI also strengthens the osteoimmune interpretation of OA by visualizing subchondral bone marrow lesions, synovitis, effusion, cartilage loss, and osteophyte formation. Bone marrow lesions are not senescence-specific, but they may identify mechanically stressed and inflammatory subchondral niches in which senescent chondrocytes, synovial cells, osteoblast-lineage cells, and immune mediators interact (90). OA senescence differs from osteoporotic senescence. Osteoporosis is dominated by systemic skeletal fragility, whereas OA is driven by local joint degeneration, mechanical stress, cartilage–bone crosstalk, and synovial inflammation. Senotherapeutic strategies for OA may therefore require local delivery and stage-specific targeting.

### 6.4 Periodontitis-related alveolar bone loss

Periodontitis is a distinct model of osteoimmune senescence because it combines microbial dysbiosis, mucosal immune activation, chronic inflammation, and alveolar bone destruction. LPS can induce premature osteocyte senescence in alveolar bone, linking bacterial products directly to senescence-associated bone pathology (83, 91).

Senescent osteocytes can worsen the periodontal microenvironment by sustaining chronic inflammation and reducing regeneration. Accumulation of senescent osteocytes exacerbates chronic inflammation and impairs regeneration in periodontal tissues (84). This supports the idea that periodontitis may represent accelerated local osteoimmune senescence. Periodontal senescence must be interpreted carefully. Periodontitis can occur in younger individuals, and senescence markers may reflect microbial burden, oxidative stress, smoking, diabetes, or chronic inflammation rather than chronological aging alone. Therefore, periodontitis is best framed as a model of inflammation-accelerated osteoimmune senescence (92). Three-dimensional imaging may be useful for linking local senescence-associated inflammation to periodontal bone destruction. CBCT can better define intrabony and furcation defects than two-dimensional radiographs in selected cases, but radiation exposure, cost, and limited evidence for routine use mean that CBCT should be used as a targeted tool for complex diagnostic or treatment-planning questions (29).

### 6.5 Impaired fracture healing and regenerative failure

Fracture healing requires coordinated inflammation, immune-cell recruitment, progenitor activation, cartilage callus formation, angiogenesis, ossification, and remodeling. Aging disrupts this sequence through impaired inflammatory resolution, macrophage dysfunction, reduced skeletal stem/progenitor competence, and persistent inflammatory signaling (93). Aged macrophages can impair fracture repair by inducing progenitor senescence. Age-related macrophage secretion of grancalcin promotes skeletal stem/progenitor cell senescence during fracture healing (17). This finding provides a direct immune-to-stem-cell mechanism for osteoimmune regenerative failure. p21-positive cells are particularly important in fracture repair. Osteochondroprogenitor cells and neutrophils expressing p21 contribute to senescence-associated signatures in fracture callus, and their targeted clearance accelerates healing (34). A related commentary has emphasized the translational implications of targeting senescent cells to improve fracture repair (94). This indicates that pathogenic senescence in repair is marker-specific and cell-type-specific. Senescence is not uniformly harmful during fracture healing. Transient senescence and inflammation may support damage sensing, immune recruitment, and tissue remodeling. Persistent or unresolved senescence may impair callus maturation and regeneration. Therefore, senotherapeutic approaches for fracture healing require careful timing and cell selectivity. Imaging-based monitoring is also central to regenerative failure. Serial radiography and CT can quantify callus mineralization and bridging, whereas MRI-based vascular or marrow assessments and functional molecular imaging may help distinguish delayed mineralization from persistent inflammatory or metabolically inactive repair niches. Such imaging endpoints could be paired with p21-positive cell mapping, macrophage-state profiling, and mechanical testing to define when senotherapy should be delivered during fracture repair.

### 6.6 Critical synthesis: shared mechanisms and disease-specific endotypes

Across these diseases, osteoimmune senescence follows a common logic but not a single pattern. Osteoporosis is often dominated by senescent osteocytes, low anabolic signaling, and marrow-niche aging. Rheumatoid arthritis-associated erosion reflects immune senescence-amplified inflammatory osteoclastogenesis. Osteoarthritis combines chondrocyte and synovial senescence with subchondral remodeling. Periodontitis represents microbial inflammation-accelerated local senescence in alveolar bone. Fracture nonunion highlights aged macrophages, persistent p21-positive repair-cell populations, and defective resolution. This disease-specific heterogeneity should guide therapy and endpoint selection. Senolytics are most rational when persistent pathogenic senescent cells are evident; senomorphics fit SASP-dominant inflammation; microbiota- or metabolite-based strategies are especially relevant when gut-derived immune tone is prominent. Imaging phenotypes such as cortical porosity, marrow osteitis, subchondral bone marrow lesions, alveolar defects, and callus maturation can help connect mechanism, disease stage, and treatment response. **Table 2** summarizes these disease-specific endotypes.

**Table 2:** Disease-specific osteoimmune senescence endotypes in aging-related bone diseases.

Disease context and representative evidence	Dominant senescent or aging-related components	Core osteoimmune mechanisms	Main skeletal consequence	Potential actionable nodes
Osteoporosis/postmenopausal osteoporosis (11, 19)	Senescent osteocytes, osteoblast-lineage cells, BMSCs, aging immune cells	Osteocyte-derived RANKL, SASP amplification, impaired osteogenesis, estrogen deficiency-related immune activation	Cortical and trabecular bone loss, marrow niche deterioration, increased fracture risk	Senolytics, anti-RANKL strategies, anabolic therapy, microbiota/metabolite modulation
Rheumatoid arthritis-associated bone erosion (14, 15)	Senescent CD4^+^CD28^−^ T cells, activated synovial fibroblasts, macrophages, osteoclast precursors	Th17/RANKL axis, inflammatory cytokine-driven osteoclastogenesis, immune senescence-amplified bone erosion	Local bone erosion, systemic bone loss, irreversible joint damage	Control of synovitis, RANKL blockade, SASP suppression, T-cell senescence targeting
Osteoarthritis-associated joint and subchondral bone degeneration (61, 81)	Senescent chondrocytes, synovial cells, subchondral bone-associated cells	SASP-driven cartilage catabolism, matrix degradation, cGAS–STING-related chondrocyte senescence, synovial inflammation	Cartilage degeneration, osteophyte formation, abnormal subchondral bone remodeling, pain and functional decline	Local senolytics, senomorphics, STING/NF-κB modulation, joint-targeted delivery
Periodontitis-related alveolar bone loss (83, 84)	Senescent osteocytes, periodontal stromal cells, microbial product-exposed bone cells	Dysbiosis-induced inflammation, LPS-driven osteocyte senescence, chronic SASP activity, osteoclast activation	Alveolar bone destruction, chronic periodontal inflammation, defective periodontal regeneration	Antimicrobial therapy, host-modulatory therapy, local senotherapy, microbiota-targeted intervention
Impaired fracture healing/regenerative failure (17, 34)	Aged macrophages, p21-positive osteochondroprogenitors, p21-positive neutrophil subsets	Macrophage-induced progenitor senescence, persistent p21-associated senescence, impaired inflammatory resolution	Delayed union, weak callus formation, reduced mechanical strength, impaired regeneration	Timed p21-positive cell targeting, macrophage reprogramming, local senotherapy
Gut–immune–bone senescence axis (21, 77)	Gut dysbiosis, altered microbial metabolites, Th17/Treg imbalance, osteoclast metabolic reprogramming	SCFA-mediated osteoclast metabolic suppression, bile acid-regulated Treg induction, barrier–immune–bone crosstalk	Systemic inflammatory bone loss, postmenopausal bone deterioration, altered osteoimmune tone	SCFAs, probiotics, bile acid metabolites, tryptophan metabolites, diet–microbiota intervention

## 7 Therapeutic targeting of osteoimmune senescence

**Figure 6:**
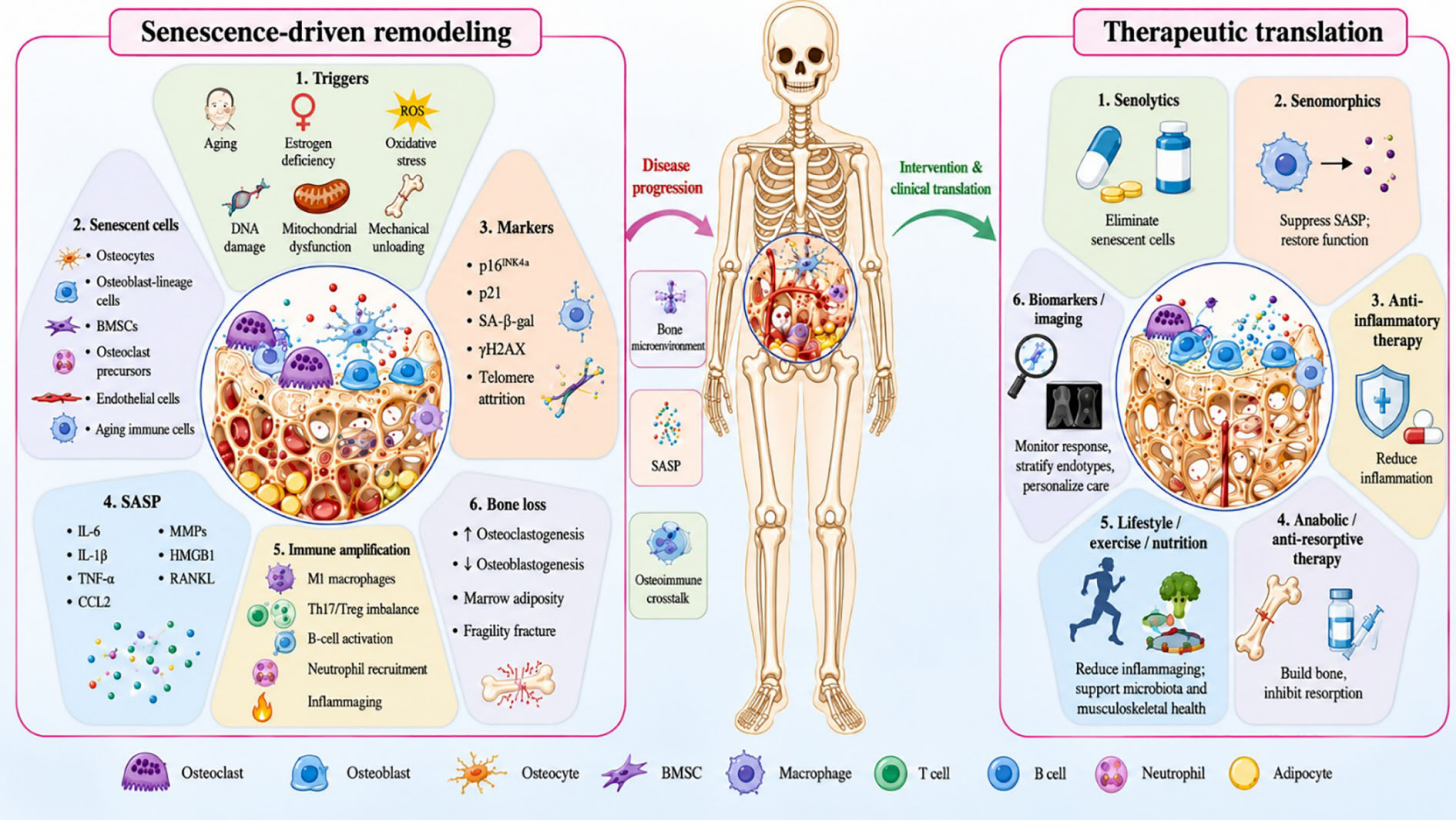
Cellular senescence-driven osteoimmune remodeling in bone loss and therapeutic translation. Aging-related estrogen deficiency, oxidative stress, DNA damage, mitochondrial dysfunction, chronic inflammation, and mechanical unloading induce senescence in osteocytes, osteoblast-lineage cells, BMSCs, osteoclast precursors, endothelial cells, and aging immune cells. SASP mediators such as IL-6, IL-1β, TNF-α, CCL2, MMPs, HMGB1, and RANKL promote macrophage polarization, Th17/Treg imbalance, B-cell activation, neutrophil recruitment, osteoclastogenesis, suppressed osteoblastogenesis, marrow adiposity, and fragility fracture. The therapeutic panel highlights senolytics, senomorphics, anti-inflammatory approaches, anabolic and anti-resorptive therapy, lifestyle/exercise/nutrition and microbiota support, and biomarker- or imaging-guided monitoring for personalized skeletal care. Representative therapeutic and translational evidence includes senescent-cell targeting, senolytic drug discovery, and the phase 2 D+Q osteoporosis trial (11, 13, 95). p16/p21-specific skeletal studies support marker-aware approaches (33, 34). JAK/NLRP3/SASP modulation supports pathway-aware senomorphic strategies (64, 65). Bone-targeted delivery, local senotherapy, and exercise effects on aging SSPCs provide examples of delivery and supportive strategies (4, 105, 106). This figure was originally created by the authors using Adobe Illustrator. BMSCs, bone marrow mesenchymal stem cells; CCL2, C-C motif chemokine ligand 2; HMGB1, high mobility group box 1; IL, interleukin; MMPs, matrix metalloproteinases; OPG, osteoprotegerin; RANKL, receptor activator of nuclear factor kappa-B ligand; SA-β-gal, senescence-associated β-galactosidase; SASP, senescence-associated secretory phenotype; TNF-α, tumor necrosis factor-alpha; Treg, regulatory T cell.

Therapeutic targeting of osteoimmune senescence is not simply an attempt to add anti-aging drugs to bone disease. Its purpose is to interrupt a pathological loop among senescent skeletal cells, dysfunctional immune compartments, chronic SASP activity, excessive osteoclastogenesis, weak osteogenesis, and poor repair resolution. Conventional antiresorptive and anabolic drugs act mainly on remodeling balance; senescence-oriented strategies target upstream cellular and inflammatory programs. The most relevant approaches include senolytics, senomorphics, immune-surveillance restoration, microbiota- or metabolite-based interventions, and bone-targeted delivery systems. Their use requires caution because senescence is context-dependent and transient senescence can participate in repair. **Table 3** summarizes the main therapeutic strategies, and **Figure 6** links senescence-inducing stressors with markers, SASP-mediated immune remodeling, bone loss, and intervention nodes.

**Table 3:** Therapeutic strategies targeting osteoimmune senescence.

Therapeutic strategy and representative evidence	Mechanistic rationale	Potential applications	Key advantages	Major limitations
Senolytics, including dasatinib plus quercetin (11, 95)	Eliminate persistent senescent cells and reduce SASP burden	Osteoporosis, postmenopausal osteoporosis, senescence-high skeletal aging	May improve both bone resorption and bone formation balance	Response may depend on baseline senescent-cell burden; off-target effects remain a concern
Marker- and cell-selective senolysis (33, 34)	Distinguish pathogenic p21-positive or p16-positive senescent populations according to disease context	Radiation-induced osteoporosis, impaired fracture healing, regenerative failure	Improves precision and may avoid eliminating reparative senescent cells	Requires validated cell-type-specific biomarkers and timing windows
SASP- and inflammation-modulating senomorphic strategies (64, 65)	Suppress harmful SASP or senescence-associated inflammatory signaling without necessarily killing senescent cells	SASP-high osteoporosis, RA-associated bone erosion, OA, periodontitis	May be safer when transient senescence is needed for repair	Senescent cells remain in tissues; chronic pathway inhibition may affect immunity
Emerging immune rejuvenation and senescent-cell surveillance restoration (18, 104)	Enhance NK-cell recognition, macrophage efferocytosis and immune clearance of senescent cells	Conceptually relevant to immune senescence-dominant skeletal aging and aging marrow niche dysfunction	Targets defective senescent-cell clearance rather than only senescent-cell production	Direct skeletal evidence remains limited; excessive immune activation may be harmful
Microbiota- and metabolite-based interventions (20, 21)	Recalibrate gut barrier function, Th17/Treg balance, osteoclast metabolism and systemic inflammaging	Postmenopausal osteoporosis, gut dysbiosis-associated bone loss, periodontitis	Links systemic metabolism, immunity and bone remodeling	Effects are strain-, dose-, diet- and host-context-dependent
Bone-targeted and local senotherapy (105, 106)	Deliver senolytics or senomorphics directly to bone, implants, periodontal defects or fracture sites	Senile osteoporosis, fracture defects, periodontal bone loss, implant-associated inflammation	Improves tissue specificity and may reduce systemic exposure	Local delivery does not guarantee cell specificity; long-term safety needs validation
Biomarker- or endotype-guided senotherapy (13)	Select patients and interventions according to senescent-cell burden, SASP profile, immune-aging phenotype or dominant disease endotype	Senescence-high osteoporosis, inflammatory bone erosion, regenerative failure, precision osteoimmunology	More suitable for heterogeneous aging-related bone diseases than unselected therapy	Requires composite biomarkers, multidimensional endpoints and prospective validation

### 7.1 Senolytics: eliminating pathogenic senescent cells

Senolytics are agents that selectively eliminate senescent cells by targeting their pro-survival pathways (95). In skeletal aging, the rationale for senolytics is supported by evidence that senescent cells accumulate in the bone microenvironment and contribute causally to age-related bone loss. Genetic and pharmacological clearance of senescent cells prevented age-related bone loss in mice, indicating that senescent cells are active drivers rather than passive markers of skeletal aging (11). However, local senolysis in aged mice only partially replicated the benefits of systemic senolysis, suggesting that spatial distribution and systemic niche effects influence therapeutic response (96). Dasatinib plus quercetin is one of the most widely studied senolytic combinations in aging-related disease models (97, 98). In aged mice, senolytic treatment reduced senescent-cell burden and improved skeletal parameters, suggesting that intermittent elimination of senescent cells can produce both anti-resorptive and pro-anabolic effects (11). This is mechanistically important because osteoimmune senescence affects both sides of bone remodeling. Preclinical studies also support the use of D+Q in estrogen-deficiency-related skeletal deterioration. Dasatinib and quercetin have been reported to ameliorate postmenopausal osteoporosis and rejuvenate bone regeneration by targeting senescent cells in experimental models (99). This finding is relevant to postmenopausal osteoporosis because estrogen deficiency can amplify immune activation, gut barrier dysfunction, and inflammatory osteoclastogenesis.

Clinical translation is better interpreted cautiously. Human D+Q feasibility has been reported in non-skeletal disease, whereas bone-specific evidence comes mainly from the phase 2 postmenopausal osteoporosis trial (13, 100). In that randomized controlled trial of intermittent D+Q in postmenopausal women, the primary endpoint of bone resorption reduction was not met in the overall cohort (13). Exploratory analyses suggested that women with higher baseline senescent-cell burden showed greater skeletal responses, including increased bone formation markers, reduced resorption markers, and improved radius bone mineral density (13). This result should not be overstated as proof of efficacy, but it is important because it suggests that senolytic response may depend on patient selection. A generic older-adult population may dilute therapeutic effects if only a subset has high senescent-cell burden or senescence-driven bone loss. Future clinical trials should therefore enrich for senescence-high endotypes using circulating p16^INK4a^ expression, SASP panels, immune-aging markers, bone turnover markers, and imaging-based skeletal phenotypes.

### 7.2 Senomorphics: suppressing harmful SASP without eliminating cells

Senomorphics, also called senostatics, aim to reduce the detrimental secretory and inflammatory features of senescent cells without necessarily inducing apoptosis. This strategy may be safer in settings where senescent cells have transient beneficial functions, such as wound healing, fracture repair, or tissue remodeling. In osteoimmune senescence, senomorphics could reduce SASP-mediated osteoclastogenesis, macrophage inflammatory priming, T-cell activation, and osteoblast suppression. JAK/STAT signaling is a representative senomorphic target. JAK inhibition reduced senescence-associated inflammatory mediators and alleviated systemic inflammation in aged mice (64). Because IL-6 family cytokines and related inflammatory signals can regulate osteoclastogenesis, osteoblast differentiation, and immune-cell activation, JAK/STAT inhibition may be relevant to osteoimmune senescence. mTOR signaling is another SASP-regulatory node. Rapamycin suppresses pro-inflammatory SASP features by regulating mTOR-dependent control of inflammatory mediator production (101). In aging bone, mTOR modulation may be relevant because excessive mTOR activity can contribute to osteoblast-lineage senescence, metabolic stress, and impaired autophagy. Metformin is also relevant because it can suppress inflammatory SASP programs and regulate cellular metabolism. Metformin inhibited the expression of multiple inflammatory SASP-related genes in senescent cells (102). In osteoblasts, loss of Men1 induced senescence through mTORC1 activation and AMPK suppression, and metformin rescued aspects of this phenotype (43).

NLRP3 inflammasome inhibition may represent another senomorphic approach. NLRP3 activation promotes IL-1β and IL-18 maturation and can connect mitochondrial dysfunction, sterile inflammation, and bone loss. Osteoblast-specific NLRP3 down-regulation protected against postmenopausal osteoporosis in experimental research, supporting the relevance of inflammasome modulation in skeletal aging (65). The advantage of senomorphics is that they may reduce chronic inflammatory damage without removing cells that still have structural or reparative functions. The limitation is that senescent cells may persist in tissues and continue to exert non-SASP pathological effects. Therefore, senomorphics may be most useful when chronic SASP is the dominant driver, whereas senolytics may be preferable when persistent senescent-cell burden is clearly pathogenic.

### 7.3 Immune rejuvenation and restoration of senescent-cell surveillance

Immune surveillance is an endogenous mechanism for limiting senescent-cell accumulation. Senescent cells can be recognized and cleared by immune cells, including NK cells, macrophages, and T-cell subsets. The immune system therefore functions not only as a contributor to osteoimmune senescence but also as a potential therapeutic tool for senescent-cell removal (103). NK cells are particularly important for senescent-cell clearance. Granule exocytosis mediates NK-cell immune surveillance of senescent cells in experimental models (18). NKG2D ligands also mediate immune surveillance of senescent cells, suggesting that activating NK-cell recognition pathways may help remove senescent-cell populations (54). Senescent cells may evade immune clearance through inhibitory checkpoint pathways. Senescent dermal fibroblasts can express HLA-E, which engages the inhibitory receptor NKG2A on NK and CD8^+^ T cells to suppress immune elimination (104). Although this mechanism has not been fully validated in bone, it provides a plausible model for why senescent skeletal cells may accumulate during aging. In osteoimmune senescence, immune rejuvenation might restore NK-cell cytotoxicity, macrophage efferocytosis, T-cell surveillance, and inflammatory resolution. This approach may be particularly relevant if aging bone is characterized by both increased senescent-cell production and reduced clearance capacity. However, direct evidence that enhancing immune surveillance removes senescent osteocytes, BMSCs, or bone marrow stromal cells remains limited. Therapeutic immune rejuvenation must also avoid excessive immune activation. Enhancing NK-cell or T-cell cytotoxicity could improve senescent-cell clearance, but it might also worsen autoimmune inflammation, tissue damage, or bone resorption in susceptible patients. This is especially relevant to rheumatoid arthritis and periodontitis, where immune activation already contributes to bone loss. A more refined approach may involve restoring immune resolution rather than simply boosting immune cytotoxicity. Macrophage efferocytosis, regulatory T-cell function, NK-cell senescent-cell recognition, and checkpoint modulation could be combined with senolytics or senomorphics. This strategy remains largely conceptual in skeletal disease, but it fits well with the osteoimmune senescence framework.

### 7.4 Microbiota- and metabolite-based interventions

Microbiota-targeted interventions may modulate osteoimmune senescence by reducing gut barrier dysfunction, systemic inflammaging, Th17/Treg imbalance, macrophage inflammatory priming, and osteoclastogenic metabolism. Probiotics prevented sex steroid deficiency-associated bone loss in mice, supporting the idea that gut microbial modulation can reduce inflammatory osteoclastogenesis (20). This finding is particularly relevant to postmenopausal osteoporosis because estrogen deficiency can connect intestinal inflammation with bone marrow immune activation. Short-chain fatty acids are among the best-supported microbial metabolites in bone regulation. SCFAs regulate systemic bone mass and protect from pathological bone loss by suppressing osteoclast metabolism (21). Butyrate and propionate can also support colonic regulatory T-cell homeostasis, linking microbial metabolism to immune tolerance (23). In osteoimmune senescence, SCFAs may act as senomorphic-like metabolites. They may not eliminate senescent cells directly, but they can reduce inflammatory propagation, support epithelial barrier integrity, promote immune regulation, and suppress osteoclastogenic metabolism. This makes them attractive for inflammation-dominant or gut dysbiosis-dominant senescence endotypes.

Bile acid metabolites provide another therapeutic avenue. Glycolithocholic acid increased circulating Treg frequency through constitutive androstane receptor signaling and alleviated postmenopausal osteoporosis (77). This indicates that microbial bile acid metabolism can be therapeutically linked to immune regulation and skeletal protection.

Tryptophan metabolites may also modify the gut–immune–bone axis. Microbial tryptophan metabolites ameliorated ovariectomy-induced bone loss by repairing intestinal AhR-mediated gut–bone signaling (78). This pathway is relevant because AhR connects microbial metabolites, epithelial barrier regulation, immune differentiation, and inflammatory tone. The major limitation is that microbiome interventions remain difficult to standardize. Probiotic effects are strain-specific, SCFA exposure depends on diet and microbial ecology, and fecal metabolite levels may not reflect bone marrow exposure. Future work needs to integrate microbial composition, functional metagenomics, targeted metabolomics, immune phenotyping, gut permeability, bone turnover markers, and bone histomorphometry.

### 7.5 Bone-targeted delivery and local senotherapy

Bone-targeted delivery is a key translational strategy because systemic senolytics may affect multiple tissues and immune compartments. Bone-targeted delivery of senolytics eliminated senescent cells and restored bone mass and microarchitecture in senile osteoporosis models (105). This result indicates that tissue-specific delivery could improve therapeutic efficacy while reducing systemic toxicity. Local senotherapy may be especially valuable in fracture healing, periodontal defects, osteoarthritis, and implant-associated bone regeneration. In a rat calvarial defect model, D+Q improved bone formation at LPS-contaminated α-tricalcium phosphate implant sites and reduced senescence markers, inflammation, macrophage accumulation, oxidative stress, and osteoclast activity (106). This supports the idea that senolytics can counteract biomaterial-related inflammatory senescence during bone repair. Biomaterial-based senotherapy can be designed to deliver senolytics, senomorphics, anti-inflammatory molecules, growth factors, or microbial metabolites in a controlled manner. Biomaterials targeting senescent cells have been proposed as a next-generation strategy for regenerating aging bone tissues (107). This direction is particularly relevant to older patients, whose bone defects often occur in a senescent and inflammatory microenvironment. Bone-targeted and local delivery strategies may also help solve the timing problem. For example, early fracture healing requires inflammatory recruitment, whereas persistent senescence later may impair callus maturation. Controlled-release systems might suppress chronic SASP or eliminate pathogenic senescent cells after the initial repair phase. The main challenge is that local delivery does not automatically guarantee cell specificity. Senolytics released into bone defects may affect progenitors, endothelial cells, macrophages, or osteoblast-lineage cells differently depending on dose, release kinetics, tissue penetration, and disease stage. Therefore, biomaterial-based senotherapy needs rigorous evaluation of cell-specific effects, mechanical outcomes, immune responses, and long-term bone quality.

### 7.6 Combination strategies with conventional bone therapies

Osteoimmune senescence is unlikely to be corrected by a single intervention. Antiresorptive agents suppress osteoclast-mediated bone loss, anabolic agents stimulate bone formation, and senotherapies target aging-related inflammatory or cellular drivers. Combining these approaches may be more effective than using senotherapy alone, particularly in patients with advanced skeletal fragility. Senolytics might be combined with anabolic therapy to create a more permissive regenerative niche. Removal of senescent cells may reduce SASP-mediated inhibition of osteoblast differentiation and allow non-senescent osteoprogenitors to repopulate bone surfaces. This idea is consistent with the observation that senescent-cell targeting in aged mice produced skeletal effects involving both reduced resorption and maintained or increased formation (11). Senomorphics may be useful as adjuncts to antiresorptives in inflammation-dominant bone disease. By suppressing chronic SASP, JAK/STAT signaling, NLRP3 activation, or mTOR-driven inflammatory programs, senomorphics may reduce immune-mediated osteoclastogenic pressure. This could be relevant in rheumatoid arthritis-associated bone loss, postmenopausal osteoporosis, periodontitis, and osteoarthritis. Microbiota-based therapies may be combined with senotherapeutics when gut barrier dysfunction or metabolite deficiency contributes to skeletal inflammation. Probiotics, dietary fiber, SCFAs, bile acid modulators, or tryptophan-metabolite strategies may reduce systemic inflammatory tone before or during senolytic intervention. This combined approach could help avoid repeated broad senolytic exposure. However, combination therapy increases complexity. Drug interactions, immune suppression, altered bone remodeling dynamics, and patient frailty must be considered. Clinical development should therefore move from empirical combinations to mechanism-based regimens guided by biomarkers of senescence burden, bone turnover, immune activation, and microbial-metabolic status.

Diet, nutrition, and lifestyle interventions may fit this combination model as anti-inflammaging or senomorphic-adjacent strategies rather than as direct senolytics. Dietary fiber can increase SCFA production and may reduce osteoclastogenic metabolism or Th17/Treg imbalance through microbial metabolites (21, 23). Exercise and mechanical loading can improve progenitor mechanosensation and partially reverse bone loss in aging-related SSPC models (4). These interventions are unlikely to replace antiresorptive or anabolic therapy in high-risk osteoporosis, but they may lower inflammatory tone, improve metabolic resilience, and increase the therapeutic window for senescence-directed approaches.

### 7.7 Timing, disease stage, and senescence endotypes

Timing is critical for senotherapy in bone disease. Senescent cells can be harmful when chronically retained, but transient senescence may contribute to tissue repair and remodeling. Studies showing possible risks of senescent-cell clearance during tissue repair have raised concerns about indiscriminate senolytic use (108). Fracture healing illustrates this issue clearly. Targeted clearance of p21-positive cells accelerated fracture repair, but the effect differed from p16-positive cell removal (34). This indicates that both timing and senescent-cell identity are essential for therapeutic design. Disease stage also matters. In early osteoporosis, senomorphics or microbiota-based strategies may reduce inflammaging before irreversible microarchitectural deterioration occurs. In established osteoporosis, senolytics may need to be combined with anabolic therapy. In fracture healing, senolytics may be more appropriate after the initial inflammatory phase if persistent senescence prevents repair resolution. The concept of senescence endotypes may improve patient selection. Patients could be classified as osteocyte senescence-dominant, immune senescence-dominant, SASP-high, gut dysbiosis-dominant, marrow adiposity-dominant, or regenerative failure-dominant. The phase 2 D+Q trial suggests that baseline senescent-cell burden may influence skeletal response, supporting the need for endotype-guided clinical trials (13).

### 7.8 Translational barriers and future directions

The largest translational barrier is biomarker uncertainty. Current markers such as p16INK4a, p21CIP1, SA-β-gal, DNA damage markers, and SASP factors are useful but not sufficiently specific when used alone. The divergence between p21-positive and p16-positive cell targeting in skeletal models highlights the need for cell-type-specific biomarkers (33). Safety is another major concern. Senolytics may affect non-senescent cells, immune memory, hematopoiesis, endothelial function, or tissue repair. The risk may be higher in older patients with frailty, multimorbidity, chronic inflammation, or polypharmacy. Therefore, intermittent dosing, local delivery, and biomarker-based selection may be safer than continuous systemic treatment. Clinical endpoints also require refinement. Bone mineral density alone may not capture osteoimmune senescence. Trials should include bone turnover markers, high-resolution skeletal imaging, fracture incidence, SASP panels, immune-aging markers, microbiome metabolites, inflammatory mediators, and functional outcomes. HR-pQCT and 18F-NaF PET/CT can be selected for microarchitecture and regional turnover (25, 28). MRI can characterize RA osteitis and OA marrow lesions (27, 90). CBCT can be used selectively for complex periodontal defects (29). This multidimensional design would better determine whether senotherapy modifies disease biology rather than only changing surrogate markers.

Several directions follow from these barriers. Human bone senescence maps need cell-type and spatial resolution. Senolytic and senomorphic strategies need disease-specific testing rather than reliance on generic aging models. Immune surveillance mechanisms require direct validation in bone. Microbiota-derived metabolites should be evaluated as immune-metabolic or senomorphic interventions, not assumed to be senolytics. Bone-targeted delivery systems must be optimized for cell specificity, release kinetics, and long-term safety. The translational value of senotherapy will ultimately depend on whether it can define treatable osteoimmune endotypes rather than offer broad anti-aging claims.

## 8 Methodological challenges and evidence gaps

Osteoimmune senescence is a useful organizing concept, but the evidence base remains uneven. Many studies rely on a small number of senescence markers, bulk tissue assays, short-term animal models, or correlations between inflammatory signals and skeletal phenotypes. These designs help generate hypotheses, yet they cannot determine which senescent cells initiate disease, which amplify pre-existing inflammation, and which transient senescence programs support repair. The field now needs sharper tools: cell-type-specific mapping, spatial localization, causal perturbation, and clinically usable endotype definitions.

### 8.1 The problem of senescence and inflammaging markers

A major limitation in osteoimmune senescence research is the absence of universal markers for both cellular senescence and inflammaging. Senescent cells are usually identified by combinations of cell-cycle arrest markers, DNA damage markers, SA-β-gal activity, SASP factors, chromatin changes, and resistance to apoptosis, but no single marker is sufficient to define senescence in all contexts (9, 10, 32). Inflammaging is even more difficult to measure because it refers to chronic, low-grade, systemic inflammation emerging from multiple tissues, microbial products, metabolic stress, immune aging, and senescent-cell SASP. Common inflammaging readouts such as IL-6, TNF-α, IL-1β, CRP, CCL2, MMPs, and innate immune activation overlap extensively with infection, obesity, autoimmunity, trauma, menopause, and mechanical stress. This explains why the concept is widely used but remains difficult to convert into a single experimental biomarker. The solution is not to search for one definitive marker, but to combine senescence markers, inflammaging panels, cell identity, spatial localization, functional perturbation, and skeletal endpoints. In bone, this means pairing p16^INK4a^, p21^CIP1^, SA-β-gal, γH2AX/53BP1, SASP factors, immune-aging markers, RANKL/OPG, sclerostin, bone turnover markers, and imaging-based microarchitecture or marrow inflammation.

These findings show that senescence markers are not interchangeable. A p16-dominant program can differ from a p21-dominant program in cell source, timing, inflammatory output, and therapeutic vulnerability. Inflammaging markers are also not interchangeable: IL-6-dominant systemic inflammation, TNF-α-driven inflammatory arthritis, microbial product-induced periodontal inflammation, and macrophage-derived inflammatory extracellular vesicles may have different skeletal consequences (7, 17, 73). The term ‘senescent cells’ therefore needs qualification by marker panel, cell type, tissue location, and functional phenotype, and the term ‘inflammaging’ should be supported by composite inflammatory, immune, metabolic, and clinical evidence. Stronger studies should combine four forms of evidence: loss of proliferative potential, stress or damage signaling, SASP or inflammatory profiling, and measurable effects on osteogenesis, osteoclastogenesis, immune activation, or tissue repair.

### 8.2 Cell-type specificity and spatial heterogeneity

Osteoimmune senescence is highly cell-type-specific. Senescent osteocytes may promote cortical bone loss through RANKL and anti-anabolic signals, whereas senescent BMSCs/SSPCs may impair osteogenesis, mechanosensation, and marrow stromal support (4, 19). Senescence-like or aged immune cells may enhance inflammatory osteoclastogenesis or reduce clearance of damaged cells, but these mechanisms are not equivalent (14, 17, 18). Recent work also cautions that immunosenescent T cells and aged macrophage-derived vesicle programs should not be collapsed into a single senescence category (7, 35). Spatial heterogeneity is equally important. Senescent cells in cortical bone, trabecular bone, marrow stroma, synovium, subchondral bone, periodontal tissues, and fracture callus may have distinct effects on local remodeling. Osteocyte-derived RANKL is particularly relevant to age-associated cortical bone loss in mice, whereas synovial or subchondral senescence may be more relevant to osteoarthritis (19, 79, 81). Periodontal senescence may be shaped by local microbial products and mucosal immune activation rather than by systemic aging alone (83).

Single-cell, spatial transcriptomic, and spatial proteomic technologies are beginning to address this limitation. Spatial transcriptomics has been used to analyze age-related defects in digit-tip bone regeneration, showing that spatially resolved approaches can reveal tissue-specific metabolic and cellular programs (109). Integrated single-cell and spatial transcriptomics can delineate cell localization and interactions in mouse fracture models, providing a template for future osteoimmune senescence studies (110, 111). Recent single-cell studies of SSPC aging and subchondral Lepr^+^ SSC senescence further illustrate how cell heterogeneity, mechanosensation, and endocrine signaling can be linked to bone loss or osteoarthritis progression (4, 79). Spatial proteomics and highly multiplexed imaging should be added to this pipeline because SASP proteins, immune checkpoints, and senescence markers are often regulated post-transcriptionally and because osteocytes, endosteal cells, vascular niches, and fracture callus cells are spatially constrained. These technologies will be most useful when combined with lineage tracing, senolytic perturbation, immune-cell functional assays, and imaging phenotypes rather than used as descriptive atlases alone.

The next step is the construction of senescence atlases for aging bone and disease-specific skeletal tissues. Such atlases should include osteocytes, osteoblast-lineage cells, osteoclast precursors, macrophages, T cells, B cells, neutrophils, endothelial cells, adipocytes, and stromal subsets. Their value will depend on linking marker expression to function, because proximity to bone surfaces, vasculature, immune clusters, or fracture callus may determine whether a senescent population is harmful, neutral, or reparative.

### 8.3 Correlation versus causality

A central evidence gap is that many studies show association rather than causation. Increased p16, p21, γH2AX, SA-β-gal, IL-6, TNF-α, or MMP expression can indicate cellular stress, inflammation, or tissue injury, but it does not prove that senescence is driving bone disease. This distinction is critical because aging-related bone diseases are also influenced by endocrine changes, mechanical loading, nutrition, medications, microbiota, vascular function, and comorbid inflammation. The strongest causal evidence comes from studies in which senescent cells are removed or senescence pathways are genetically manipulated. Targeting senescent cells prevented age-related bone loss in mice, supporting a causal role for senescence in skeletal aging (11). Local clearance of senescent cells attenuated post-traumatic osteoarthritis, showing that senescent cells can actively promote joint degeneration (81). However, causal evidence is not equally strong across all diseases and cell types. Osteocytes and BMSCs have relatively strong support as skeletal senescence sources, but the direct causal roles of senescent B cells, NK cells, endothelial cells, and marrow adipocytes in aging bone remain less established. Similarly, senescent T cells have direct osteoclastogenic evidence in rheumatoid arthritis, but their role in primary age-related osteoporosis requires further testing (14).

Causality will require more decisive experimental designs, including cell-specific genetic clearance, lineage tracing, adoptive transfer of senescent immune cells, SASP neutralization, senolytic rescue experiments, and time-resolved disease models. Evidence is strongest when senescent-cell removal reduces pathology, reintroduction of the candidate cell population restores the phenotype, and specific SASP mediators reproduce disease activity after senolysis. Timing also matters. Senescence can initiate disease in one context, amplify inflammation in another, and impair repair only after resolution fails.

### 8.4 Animal model limitations

Animal models are indispensable for mechanistic studies, but they do not fully reproduce human osteoimmune aging. The ovariectomized rodent model is widely used to study postmenopausal osteoporosis, but it primarily models acute estrogen deficiency rather than the full complexity of natural aging. Reviews of osteoporosis models emphasize that model choice must consider species, skeletal site, age, sex, and the specific disease mechanism under investigation (112). OVX models are useful for studying estrogen-deficiency-induced bone loss, but they may overrepresent high-turnover bone resorption and underrepresent chronic immunosenescence, marrow niche aging, frailty, and multimorbidity. Animal models for postmenopausal osteoporosis are generated by ovariectomy, but bone loss in humans is also shaped by aging, muscle decline, nutrition, inflammation, and endocrine-metabolic interactions (113). Therefore, OVX findings should not be generalized automatically to all forms of aging-related osteoporosis. Natural aging models provide better biological relevance but require longer experimental timelines and are affected by strain, sex, housing environment, microbiota, diet, and baseline skeletal phenotype. Aging affects fracture healing through immune senescence and increased systemic inflammatory status, which may not be captured in young OVX animals (114). Studies comparing young and geriatric mice also show age-dependent differences in fracture healing that require direct modeling of advanced age (115).

Microbiome-related models create additional challenges. Gut microbiota composition differs by facility, cage, diet, antibiotic exposure, vendor, and animal strain. A microbiota-dependent bone phenotype in one facility may not reproduce under different microbial conditions. This is especially important for studies linking dysbiosis, SCFAs, bile acids, tryptophan metabolites, immune activation, and bone loss. Disease-specific models also have limitations. Collagen-induced arthritis and other inflammatory arthritis models can illuminate immune-driven bone erosion, but they do not fully recapitulate human rheumatoid arthritis chronicity, treatment exposure, autoantibody heterogeneity, and age-related immune remodeling. Surgical OA models capture post-traumatic degeneration, but they may not reflect slowly progressive age-associated OA. Periodontitis models can reproduce microbial inflammation, but they may not capture decades of human periodontal aging.

### 8.5 Human translation and biomarker development

Translation from animal models to human osteoimmune senescence requires clinically usable biomarkers. Candidate markers include circulating SASP factors, p16INK4a expression in immune cells, p21-related signatures, DNA damage markers, immune-aging phenotypes, bone turnover markers, microbial metabolites, and imaging-based skeletal measures. However, none of these markers alone is sufficient to identify disease-driving senescence in bone. Clinical trials already suggest that patient selection may be essential. In a phase 2 trial of intermittent dasatinib plus quercetin in postmenopausal women, the overall primary endpoint was not met, but exploratory analyses suggested greater skeletal responses in participants with higher senescent-cell burden (13). This result supports the idea that senotherapy may require senescence-high endotype enrichment rather than unselected enrollment. Bone mineral density alone may be an insufficient endpoint for osteoimmune senescence trials. BMD does not directly measure SASP burden, immune dysfunction, marrow inflammation, osteocyte senescence, cortical porosity, trabecular microarchitecture, or regenerative capacity. Future trials should combine BMD with HR-pQCT-based microarchitecture and 18F-NaF PET/CT-based turnover readouts when relevant (25, 28). MRI-based marrow or joint assessment can support inflammatory and adiposity-related endotyping (26, 27, 90). CBCT may be useful when periodontal defects are a key endpoint (29). These should be paired with bone turnover markers, circulating SASP panels, immune phenotyping, microbiome/metabolite profiling, and fracture-related outcomes.

The field also needs biomarkers that can separate senescence burden from general inflammation. IL-6, TNF-α, IL-1β, and MMPs are commonly associated with SASP, but they can also arise from infection, autoimmunity, obesity, trauma, or mechanical stress. Therefore, senescence biomarkers are better interpreted alongside cell source, clinical context, tissue localization, and response to senescence-targeted intervention. A practical clinical challenge is tissue accessibility. Human osteocytes, marrow stromal cells, and fracture callus are difficult to sample repeatedly. Blood-based markers are easier to measure but may not reflect local skeletal senescence. This mismatch may partly explain why systemic senescence signatures do not always predict bone-specific outcomes. Imaging biomarkers are therefore best treated as complementary rather than merely descriptive. HR-pQCT can quantify cortical and trabecular deterioration (25). MRI can identify marrow fat or osteitis (26, 27). 18F-NaF PET/CT can capture regional bone metabolic activity (28), and CBCT can define local periodontal bone morphology (29). When these imaging features are analyzed alongside blood-based SASP factors, immune-aging profiles, and bone turnover markers, they may help distinguish senescence-high skeletal deterioration from nonspecific inflammation or mechanical degeneration.

### 8.6 Endpoint heterogeneity across diseases

Osteoimmune senescence cannot be evaluated with one universal endpoint. Osteoporosis studies may prioritize BMD, HR-pQCT-derived microarchitecture, marrow adiposity, bone turnover, and fracture risk. Rheumatoid arthritis-associated bone erosion requires imaging of erosions, synovitis, osteitis or bone marrow edema, inflammatory markers, and osteoclastogenic cytokines. Osteoarthritis studies require cartilage structure, pain, synovitis, subchondral bone remodeling, MRI bone marrow lesions, and function. Periodontitis studies require alveolar bone loss, periodontal inflammation, microbial profiles, CBCT-defined defects in selected cases, and tissue regeneration endpoints. Fracture-healing studies require callus formation, mechanical strength, mineralization, vascularization, and time to union. This endpoint heterogeneity complicates comparison across disease settings. A senolytic that improves fracture repair may not necessarily improve systemic osteoporosis. A senomorphic that reduces synovial inflammation may not restore bone formation. A microbiota-derived metabolite that suppresses osteoclastogenesis may not be sufficient to improve cartilage degeneration or callus biomechanics. Therefore, each osteoimmune senescence endotype should have disease-specific endpoints. Osteocyte-dominant osteoporosis may require cortical porosity, osteocyte RANKL, sclerostin, and bone turnover readouts. Immune-dominant RA bone erosion may require T-cell senescence markers, RANKL expression, erosion progression, and inflammatory activity. Regenerative failure may require progenitor function, macrophage state, p21-positive callus cells, and mechanical repair strength. A critical future direction is to develop composite endpoint panels. These panels should combine senescence markers, immune markers, bone remodeling markers, tissue imaging, radiomics-ready structural features, and functional outcomes. Such designs would be more informative than relying on a single molecular marker or a single skeletal endpoint.

### 8.7 Distinguishing harmful, neutral, and beneficial senescence

Not all senescence is harmful. Senescence can contribute to wound healing, tissue remodeling, tumor suppression, immune recruitment, and damage containment. Cellular senescence in bone has been discussed as a process with both detrimental chronic effects and potentially beneficial acute functions (8). This duality is particularly important for fracture healing and tissue regeneration. The timing of senescence is therefore a major unresolved issue. Early transient senescence may coordinate repair signals, whereas persistent senescence may block regeneration and sustain inflammation. In fracture repair, evidence that p21-positive cell clearance accelerates healing must be interpreted in relation to timing, cell identity, and repair stage (34). Broad senescent-cell clearance without temporal control might remove cells that contribute to early repair. Senolytic studies should therefore distinguish acute adaptive senescence from chronic maladaptive senescence. This requires time-course experiments, cell-fate tracking, and stage-specific intervention. A senolytic administered before injury, during early inflammation, during callus formation, or during remodeling may produce different outcomes. This issue is also relevant to osteoarthritis and periodontitis. Local senescent-cell clearance may reduce chronic joint degeneration, but the same strategy may have different effects in early injury, active inflammation, or late-stage tissue destruction. A disease-stage-specific framework is needed before senolytics can be safely generalized across bone-related diseases.

### 8.8 Need for standardization and reproducibility

Osteoimmune senescence studies require greater methodological standardization. Researchers should report animal age, sex, strain, skeletal site, housing conditions, diet, microbiota status, mechanical loading, endocrine manipulation, and comorbid disease context. These variables can influence bone mass, immune phenotype, senescence markers, microbial metabolites, and response to therapy. Senescence assays also need standardized reporting. Studies should specify marker combinations, detection methods, antibody validation, tissue processing, cell-type identification, and quantification strategy. A study based on p16 immunostaining alone should not be interpreted with the same confidence as one combining p16, p21, DNA damage, SASP profiling, spatial localization, and functional rescue. Microbiome-related studies require additional controls. Antibiotic treatment, germ-free status, fecal microbiota transplantation, diet composition, cage effects, and facility microbiota should be clearly reported. Functional readouts should include metabolites and immune phenotypes, not only taxonomic abundance. Senotherapy studies should also report dosing schedule, timing relative to disease stage, off-target toxicity, immune effects, bone turnover dynamics, and long-term skeletal quality. This is especially important because senolytics may have different effects depending on whether disease is dominated by osteocyte senescence, immune senescence, SASP propagation, gut dysbiosis, or regenerative failure.

### 8.9 Future research priorities

The main priorities are straightforward but technically demanding: map senescent-cell states in human aging bone; define endotypes based on senescence burden, SASP activity, immune aging, microbial-metabolic status, marrow adiposity, and repair capacity; prove causality in disease-specific models; and develop biomarkers that predict response to senolytics, senomorphics, microbiota interventions, or immune-recalibrating therapies. Mechanism-based clinical trials should then test defined interventions in defined endotypes, using multidimensional endpoints rather than BMD alone.

### 8.10 Critical synthesis

The central challenge is to move from descriptive association to mechanistic precision. There is strong evidence that senescent cells contribute to age-related bone loss, post-traumatic osteoarthritis, and impaired fracture healing, but there is still no complete map of which senescent cells matter most in each disease context (11, 81). The divergent effects of p16-positive and p21-positive cell targeting show that senescence is not one biological entity (33, 34). The decisive question is therefore not whether senescence exists in aging bone, but which cell population drives which skeletal phenotype at which stage.

## 9 Future perspectives: toward precision senotherapeutics in osteoimmunology

The next phase of osteoimmune senescence research must narrow a broad concept into testable clinical programs. The field no longer needs to show only that senescent cells accumulate in aging bone. It needs to identify which senescent cell populations are pathogenic, how immune circuits sustain them, which skeletal compartments are affected, which imaging phenotypes reflect tissue-level injury, and which patients are likely to benefit from senescence-directed therapy. This shift requires integration of osteoimmunology, senescence biology, spatial multi-omics, microbiome science, imaging, and mechanism-based trial design.

### 9.1 Identify actionable osteoimmune senescence endotypes

Clinical diagnosis alone is too coarse for precision senotherapy. The endotypes proposed here should be treated as working hypotheses rather than validated clinical categories. Osteoporosis, rheumatoid arthritis-associated erosion, osteoarthritis, periodontitis, and delayed fracture healing all involve senescence-related biology, but the dominant pathogenic program differs and the evidence strength is uneven. Potential endotypes include osteocyte senescence-dominant cortical bone loss, BMSC or osteoblast-lineage exhaustion, immune senescence-driven osteoclastogenesis, SASP-high inflammatory remodeling, gut dysbiosis-associated inflammaging, and regenerative failure with persistent p21-positive repair-cell populations. This classification would explain why a senolytic, senomorphic, immune-recalibrating intervention, or microbial metabolite strategy might work in one setting but not another. It also keeps therapeutic interpretation tied to stage, because early inflammatory remodeling, established architectural damage, and failed repair require different intervention windows (11, 13, 34).

### 9.2 Link biomarkers with imaging phenotypes

Clinically useful biomarkers will probably be composite rather than single-marker readouts. Circulating SASP factors, p16INK4a or p21CIP1 expression in immune cells, immune-aging phenotypes, bone turnover markers, microbial metabolites, and osteocyte-derived signals each capture part of the biology, but none alone identifies disease-driving skeletal senescence. Imaging can strengthen these markers by anchoring them to tissue-level pathology. HR-pQCT-derived cortical porosity and 18F-NaF PET/CT-derived regional turnover can capture structural and metabolic skeletal readouts (25, 28). MRI-detected marrow adiposity or osteitis and CBCT-defined alveolar defects can help define local disease phenotypes (26, 29). The phase 2 D+Q trial in postmenopausal women further suggests that baseline senescent-cell burden may influence skeletal response, supporting biomarker-guided enrollment rather than unselected testing (13).

### 9.3 Design cell-selective and stage-specific senotherapeutics

Senotherapeutic design should move away from broad senolysis. The divergent skeletal effects of p16-positive and p21-positive cell targeting show that senescent-cell identity matters (33, 34). Osteocyte-targeted strategies may be relevant to cortical bone loss, whereas p21-positive cell clearance may be more relevant to impaired fracture repair. Senomorphics may be preferable when chronic SASP, rather than cell burden itself, is the dominant driver. JAK/STAT, mTOR, NF-κB, NLRP3, and cGAS-STING pathways are plausible targets, but pathway inhibition must preserve host defense and repair. Bone-targeted or local delivery systems could improve the therapeutic window in osteoporosis, fracture defects, periodontal bone loss, implant-associated inflammation, and osteoarthritis (105).

### 9.4 Build mechanism-based clinical trials

Future trials need to test a defined intervention in a defined osteoimmune endotype with endpoints that show target engagement, tissue response, and clinical relevance. For osteoporosis, this means combining BMD with HR-pQCT microarchitecture, bone turnover markers, SASP panels, and immune-aging profiles. For rheumatoid arthritis, MRI osteitis, erosion progression, synovitis, and osteoclastogenic cytokines may be more informative. For fracture healing, timing relative to inflammation, callus formation, and remodeling is essential. For periodontitis and osteoarthritis, local delivery and local imaging or tissue endpoints may be more informative than systemic measures alone. Safety monitoring must address hematopoiesis, endothelial function, infection risk, immune memory, wound healing, and polypharmacy in older adults. The practical goal is not a general anti-aging therapy for bone, but a precision osteoimmunology platform that matches the dominant senescence program to a measurable intervention strategy. Overall, osteoimmune senescence offers a strong framework for reinterpreting aging-related bone diseases as immune-skeletal disorders driven by senescent-cell burden, SASP propagation, immune remodeling, microbial-metabolic dysfunction, imaging-detectable tissue damage, and impaired repair. Its translational value will depend on whether future work can convert this framework into validated endotypes, deployable biomarker panels, and targeted therapies that improve bone strength, reduce inflammatory damage, and restore regenerative competence.
